# Long CAG Repeat Sequence and Protein Expression of Androgen Receptor Considered as Prognostic Indicators in Male Breast Carcinoma

**DOI:** 10.1371/journal.pone.0052271

**Published:** 2012-12-14

**Authors:** Yan-Ni Song, Jing-Shu Geng, Tong Liu, Zhen-Bin Zhong, Yang Liu, Bing-Shu Xia, Hong-Fei Ji, Xiao-Mei Li, Guo-Qiang Zhang, Yan-Lv Ren, Zhi-Gao Li, Da Pang

**Affiliations:** 1 Department of Breast Surgery, The Third Affiliated Hospital of Harbin Medical University, Harbin, China; 2 Department of Pathology, The Third Affiliated Hospital of Harbin Medical University, Harbin, China; 3 Department of Genomics, Heilongjiang Institute for Cancer Research, Harbin, China; Health Canada, Canada

## Abstract

**Background:**

The androgen receptor (AR) expression and the CAG repeat length within the AR gene appear to be involved in the carcinogenesis of male breast carcinoma (MBC). Although phenotypic differences have been observed between MBC and normal control group in AR gene, there is lack of correlation analysis between AR expression and CAG repeat length in MBC. The purpose of the study was to investigate the prognostic value of CAG repeat lengths and AR protein expression.

**Methods:**

81 tumor tissues were used for immunostaining for AR expression and CAG repeat length determination and 80 normal controls were analyzed with CAG repeat length in AR gene. The CAG repeat length and AR expression were analyzed in relation to clinicopathological factors and prognostic indicators.

**Results:**

AR gene in many MBCs has long CAG repeat sequence compared with that in control group (*P* = 0.001) and controls are more likely to exhibit short CAG repeat sequence than MBCs. There was statistically significant difference in long CAG repeat sequence between AR status for MBC patients (*P* = 0.004). The presence of long CAG repeat sequence and AR-positive expression were associated with shorter survival of MBC patients (CAG repeat: *P* = 0.050 for 5y-OS; *P* = 0.035 for 5y-DFS AR status: *P* = 0.048 for 5y-OS; *P* = 0.029 for 5y-DFS, respectively).

**Conclusion:**

The CAG repeat length within the AR gene might be one useful molecular biomarker to identify males at increased risk of breast cancer development. The presence of long CAG repeat sequence and AR protein expression were in relation to survival of MBC patients. The CAG repeat length and AR expression were two independent prognostic indicators in MBC patients.

## Introduction

Male breast carcinoma (MBC) is an uncommon disease, accounting for less than 1% of all cases of breast carcinoma. Hormonal factors are specifically important in carcinogenesis of male breast. Androgens, like dihydrotestosterone, restrain tissue growth in the breast thereby exerting anti-mitogenic effects [1]. The androgen receptor (AR) activity modulates breast tissue growth inhibition. Less is known about AR genetic influences and AR protein expression in MBC carcinogenesis.

Germline mutations in the AR gene have also been suggested to predispose to MBC [2]. A region within exon 1 of the gene coding for the AR (located on chromosome Xq11-12) is highly polymorphic three nucleotides repeat and contains a variable number of CAG repeats. The transactivational ability of the AR is inversely related to its length [1]. In breast tissue, a long AR will be functionally weaker and worse at restraining tissue growth. Within the AR the highly polymorphic region of glutamine repeats called the CAG repeat sequence is a prime candidate as a risk modulator.

The AR has biological and therapeutic significance in prostate cancer [3]. ARs are also expressed in 70% to 90% of primary or metastatic MBC patients. It is presumed that MBC is biologically similar to female breast carcinomas (FBC). However, there are differences between the two: a higher frequency of expression of ER (81%) and progesterone receptor (PR) (74%) is in MBC [4]. The molecular mechanisms of AR signaling pathway in breast carcinoma biology are not clearly understood. AR may have some essential role in MBC, but the clinical significance of AR expression has not been well characterized.

The aims of the present study were to: (a) investigate whether increased length of the CAG repeat sequence in the AR gene is associated with the development of MBC. (b) compare CAG repeat lengths with clinicopathological features and hormone receptor expression; (c) evaluate prognostic significance of AR (CAG)n repeats length and AR expression; and (d) present a new opinion of endocrine therapy (anti androgenic therapy) for MBC patients.

## Materials and Methods

### Study Population

Controls and patients in this study came from two hospitals, The Third Affiliated Hospital of Harbin Medical University (the 3rd AHMU) and the JingWei Affiliated Hospital of Harbin Medical University (the JW AHMU) in Heilongjiang Province northeast China.

### Controls

Eighty blood samples were collected from blood donors at the JW AHMU from January to July of 2012. Samples were from residual whole blood donations of men who did not have a personal history of breast or prostate cancer or a family history of MBC. The study was approved by the Institutional Review Boards of Harbin Medical University and the Heilongjiang Province Cancer Society.

### Cases

MBC patient records were obtained from 175 patients at the 3rd AHMU and 33 patients at the JW AHMU; these 208 subjects represented all male patients with breast cancer seen at the two institutions during the period of 1990–2007. Patients’ medical records were retrieved and the following information was abstracted from them: histopathologic and treatment characteristics, such as tumor size, histological grade, hormone receptor status, and lymph node involvement. Of the 208 subjects, tissues were obtained from 85 patients, 76 from the 3rd AHMU, and 9 from the JW AHMU; AR (CAG)n repeats length was determined for 77 and 8, respectively. No patient received any therapy before surgery. Ethical approval for the study was obtained through the Heilongjiang Regional Ethics Committee.

### DNA Extraction and PCR-based GeneScan Analysis

Formalin-fixed paraffin-embedded (FFPE) tumor tissues were obtained from 85 Chinese men with breast cancer. Hematoxylin and eosin staining and analysis by tow pathologists allowed for differentiation of normal tissue from tumor tissue for 81 cases. 31 blood samples were obtained from our tissue bank in these 81 cases. Microdissection of normal tissue from the tumor blocks allowed for working samples to represent the true genome in MBC patients. DNA was isolated according to a protocol specialized for FFPE tissues provided by OMEGA (Tissue DNA Kit, make in USA). For the control series, DNA extraction was from whole blood using standard protocol from AXYGEN (Blood Genomic DNA Kit, Union City, CA94587 USA).

### AR (CAG) n Repeats Length Determination

PCR was used to amplify the AR gene CAG repeat region. Primers AR-F 5′-TGCGCGAAGTGATCCAGAACC-3′ and AR-R 5′-CTCATCCAGGACCAGGTAGCC-3′ were chosen to amplify a region containing the repeat polymorphism of about 250 base pairs [5]. The forward primer was fluorescently labeled with 6-carboxy-fluorescine (FAM). 100 ng of DNA was amplified in a reaction mix containing 1 µl of each primer at 10 pmol, 0.8 µl 50 mM MgCl_2_, 0.2 µl Platinum® Taq DNA polymerase (Life technologies Invitrogen, USA), 10 µl of Gene Amp Fast PCR Mix from ABI (Applied Biosystems, USA), and distilled water to bring to final volume to 25 µl. PCR was performed according to a standard protocol (modified from Gilbert SF [6]); starting with 94°C for 2 min; followed by 30 cycles of 94°C denaturation for 30 s, 55°C annealing for 30 s, and 72°C extension for 1 min; ending with a final extension at 72°C for 30 min. Digital images of fluorescent gel data were acquired using Data Collection Software and analyzed using GeneScan Software (both from Applied Biosystems). The results were imported into the Genetyper Software (Applied Biosystems) for further analysis.

### Immunohistochemistry and AR Expression Determination

Breast cancer tissue microarrays were prepared and immunohistochemically stained for AR expression. A rabbit polyclonal to AR antibody (ab74272; dilution 1∶100; ABcam) was used to evaluate AR expression. Sections from FFPE specimens were cut at 4 µm thick, were transferred onto adhesive glass slides, and incubated for 30 min at 62°C. Sections were dewaxed and rehydrated according to routine procedure, then pretreated for antigen retrieval in Ethylene Diamine Tetraacetic Acid (EDTA) buffer (pH 8.0) in a microwave oven for 20 min. Endogenous peroxidase activity was blocked using 3% H_2_O_2_ for 30 min. After incubation with the primary antibodies, sections were incubated at 4°C overnight with primary antibodies. After a washing, sections were incubated with biotinylated anti mouse immunoglobulin for 30 min. Reaction products of all markers under investigation were visualized using 3, 3′-diaminobenzidine as chromogen. Sections were counterstained with hematoxylin, dehydrated through graded ethanol, cleared in xylene, and mounted. The sections were observed under a light microscope. Human prostate carcinoma tissue (from other patients) was used for positive control for AR. PBS was used to replace the primary antibody and served as the negative control.

The criteria for AR positivity were based on the intensity and percentage of tumor cells showing expression. The intensity was graded as negative, weak, moderate or strong. Tumors that had more than 10% of cells exhibiting weak, moderate or strong intensity of expression were considered positive.

### Statistical Analysis

The relationship among clinicopathological features, sex hormone receptor status, and CAG repeat lengths was analyzed with a chi-square test. Overall survival (OS) was calculated from the date of surgery until death or the date patients were last known to be alive. The disease-free survival (DFS) was calculated from the date of surgery until relapse or the date patients were last known to be alive. Univariate survival analyses were based on Kaplan-Meier method. The relative importance of multiple prognostic factors on survival was estimated using the Cox proportional hazard regression model. Statistical analysis was performed using SPSS 16.0 statistical software (SPSS Inc., Chicago, USA). *P*<0.05 was considered to be significant.

## Results

### Comparison of CAG Repeat Length between MBC and Control Group

We detected CAG repeat length of bloods and tumor tissues in 31 MBC patients. The test results of two paired samples were the same in each patient (Figure S1).

**Figure 1:**
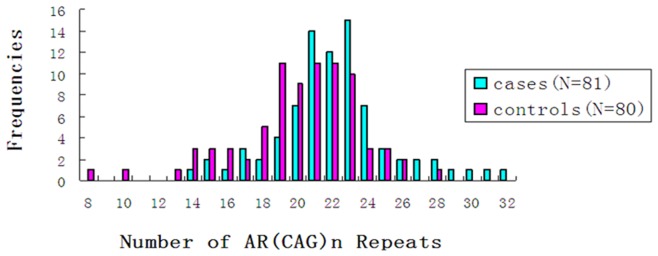
Distribution of CAG repeat length among the MBC patients and control groups.

The lengths of the PCR products obtained varied between 206 bp and 278 bp (corresponding to 8 CAG repeats and 32 CAG repeats, respectively). The median number of CAG repeats for 81 MBC patients was 20.5. This study used allele lengths of <21 and ≥21 as “short” and “long” CAG repeat sequence cut-points. Fig. 1 illustrates the CAG repeat lengths distribution among the MBC patients and control groups. This can be intuited in Fig. 1, alleles in the control group consisted of 28 or less CAG repeats, whereas 6 MBC patients had alleles with 28–32 repeats and the majority of CAG repeat lengths to be within 14–28 repeats in MBC group. Table 1 showed the MBC patients and controls had unequal proportions of subjects with long CAG repeat sequence (MBC patients: 59.8%, Controls: 40.2%, *P* = 0.001). It was thus clear that controls were more likely to exhibit short CAG repeat sequence than MBC patients (MBC patients: 33.9%, Controls: 67.1%).

**Table 1:** Comparison of CAG repeat length in normal population and MBC patients.

	No. of CAG Repeat length		*P*
	Short (%)	Long (%)	
**MBCs**	20 (24.7%)	61 (75.3%)	**.001**
**Controls**	39 (48.7%)	41 (51.3%)	

### Analysis of AR Protein Expression in MBC

**Figure 2:**
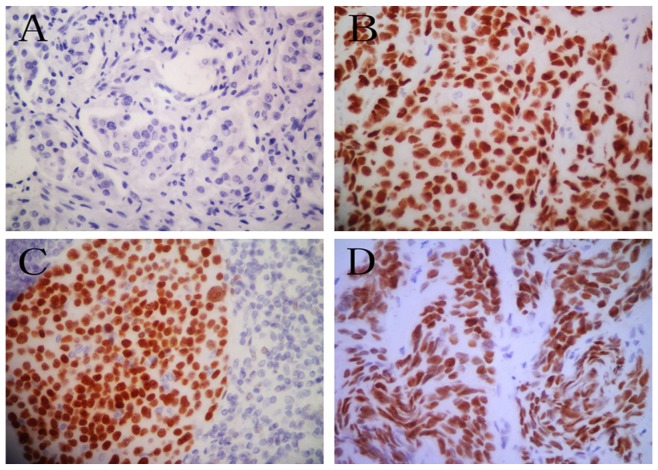
Immunohistochemical staining of the AR. A. negative staining; B. strong positive staining; C. immunoreactivity in axillary metastatic lymph node; and D. immunoreactivity in chest wall recurrence.

AR protein was expressed, mainly in cell nuclei with weak, moderate and strong intensity. The AR staining results were as follows: 14 cases showed negative and 67 cases were positive inmunostaining (Fig. 2A and B). The positive rate for AR expression was 82.7%. The positive rates for axillary metastatic lymph node and chest wall recurrence were 92.0%, and 90.5%, respectively (Fig. 2C and D).

Table 2 showed the CAG repeat lengths between AR-negative expression and AR-positive expression in MBC patients. There was statistically significant difference in long CAG repeat sequence between AR-negative and AR-positive for MBC patients (*P* = 0.004).

**Table 2:** Comparison of CAG repeat length between AR (–) and AR (+) in MBC patients.

	No. of CAG Repeat length		*P*
	Short	Long	
**AR (–)**	8 (40%)	6 (10%)	**.004**
**AR (+)**	12 (60%)	55 (90%)	

The CAG repeat lengths and AR expression was analyzed in relation to clinical, pathological, and biochemical features such as age, stage of disease, tumor size, lymph node involvement, histological grade, and sex hormone receptor expression (Table 3). The percentage of long CAG repeat sequence was higher in bigger tumors (>2 cm) (*P* = 0.033). AR expression was related to ER (*P* = 0.023) and PR expression (*P* = 0.024; Table 3).

**Table 3:** AR expression and AR (CAG) n repeat length in MBC patients in relation to clinical and pathological features.

Factor	N	No. of AR (n = 81)			No. of CAG Repeat length		
		AR (–)	AR (+)	*P*	Short	Long	*P*
**Age**				**.301**			**.576**
** <60**	37	5 (36%)	32 (48%)		9 (45%)	28 (46%)	
** ≥60**	44	9 (64%)	35 (52%)		11 (55%)	33 (54%)	
**Pathological T stage**				**.377**			**.033**
** T1 (**≤**2 cm)**	18	4 (29%)	14 (21%)		8 (40%)	10 (16%)	
** T2-4 (>2 cm)**	63	10 (71%)	53 (79%)		12 (60%)	51 (84%)	
**Pathological N stage**				**.540**			**.528**
** N0**	31	5 (36%)	26 (39%)		8 (40%)	23 (38%)	
** N1-3**	50	9 (64%)	41 (61%)		12 (60%)	38 (62%)	
**Histological grade**				**.233**			**.205**
** G1**	10	3 (21%)	7 (10%)		4 (20%)	6 (10%)	
** G2-3**	71	11 (79%)	60 (90%)		16 (80%)	55 (90%)	
**ER**				**.023**			**.174**
** ER (–)**	20	7 (50%)	13 (19%)		7 (35%)	13 (21%)	
** ER (+)**	61	7 (50%)	54 (81%)		13 (65%)	48 (79%)	
**PR**				**.024**			**.421**
** PR (–)**	25	8 (57%)	17 (25%)		7 (35%)	18 (30%)	
** PR (+)**	56	6 (43%)	50 (75%)		13 (65%)	43 (70%)	
**HER2**				**.314**			**.475**
** HER2 (–)**	59	9 (64%)	50 (75%)		14 (70%)	45 (74%)	
** HER2 (+)**	22	5 (36%)	17 (25%)		6 (30%)	16 (26%)	
**Ki-67**				**.131**			**.412**
** Ki-67<10%**	17	5 (36%)	12 (18%)		5 (25%)	12 (20%)	
** Ki-67≥10%**	64	9 (64%)	55 (82%)		15 (75%)	49 (80%)	
**Operation**				**.575**			**.371**
** Single mastectomy**	28	5 (36%)	23 (34%)		8 (40%)	20 (33%)	
** MRM** *	53	9 (64%)	44 (66%)		12 (60%)	41 (67%)	
**Chemotherapy**				**.418**			**.524**
** Yes**	51	8 (57%)	43 (64%)		13 (65%)	38 (62%)	
** No**	30	6 (43%)	24 (36%)		7 (35%)	23 (38%)	
**Hormone therapy**				**.208**			**.525**
** Yes**	47	10 (71%)	37 (55%)		12 (60%)	35 (57%)	
** No**	34	4 (29%)	30 (45%)		8 (40%)	26 (43%)	

* MRM: Modified radical mastectomy.

### Survival Analysis of CAG Repeat Length and AR Expression in MBC

Table 4 showed OS and DFS were analyzed according to CAG repeat lengths, AR expression and clinicopathological parameters. The 5-year OS for all 81 patients was 58.0% at 5 years, 70.0% versus 49.2% for short CAG repeat sequence and long CAG repeat sequence patients, respectively (*P* = 0.050). The 5-year DFS for the entire group was 45.7%. The 5y-DFS, similarly to 5y-OS, was found to differ with respect to CAG repeat length and was significantly decreased in long CAG repeat sequence patients (60% versus 39.3%, *P* = 0.035). The 5y-OS and 5y-DFS were significantly decreased for MBC patients with tumors staining positively for AR (*P* = 0.048 for 5y-OS; *P* = 0.029 for 5y-DFS). Furthermore, the 5y-OS and 5y-DSF rates were also significantly decreased for MBC patients in lymph node metastases. The 5y-OS was 80.6% for lymph node negative group and 44% for lymph node metastases (*P* = 0.001). The 5y-DSF was 71.0% for lymph node negative and 30% for lymph node metastases (*P* = 0.001). Shorter 5y-DFS was correlated with increasing Ki-67 (*P* = 0.048 for 5y-DFS). There was no difference in 5y-OS rate with respect to Ki-67 status (*P* = 0.98).

**Table 4:** Associations of CAG repeat lengths, AR expression, and clinicopathological features with survival of MBC patients.

Factor	N	5-y survival rate (%)		Median survival (mos)		*P*	
		5y-OS	5y-DFS	5y-OS	5y-DFS	5y-OS	5y-DFS
**Total**	81	58.0	45.7	49.5	42.0		
**Age**							
** <60**	37	59.5	45.9	49.9	41.9	.812	.977
** ≥60**	44	56.8	45.5	49.1	42.1		
**T stage**						.223	.095
** pT1**	18	72.0	66.7	51.2	46.5		
** pT2-4**	63	54.0	39.7	48.8	40.7		
**N stage**						.001	.001
** pN0**	31	80.6	71.0	56.0	50.8		
** pN1-3**	50	44.0	30.0	45.4	36.6		
**Histological grade**						.892	.822
** G1**	10	60.0	50.0	50.7	43.1		
** G2-3**	71	57.7	45.1	49.3	41.8		
**ER**						.219	.165
** ER (–)**	20	50.0	35.0	41,4	36.0		
** ER (+)**	61	60.7	49.2	52.0	44.0		
**PR**						.293	.646
** PR (–)**	25	68.0	52.0	48.5	41.5		
** PR (+)**	56	53.6	42.9	49.8	42.2		
**HER2**						.360	.389
** HER2 (–)**	59	54.2	42.4	49.6	41.1		
** HER2 (+)**	22	68.2	54.5	48.9	44.4		
**Ki-67**						.098	.048
** Ki-67<10%**	17	75.0	62.5	56.0	55.2		
** Ki-67≥10%**	64	53.8	40.0	46.7	38.5		
**Operation**						.701	.841
** Single mastectomy**	28	56.7	41.1	46.8	43.3		
** MRM** *	53	58.6	42.3	51.0	45.7		
**Chemotherapy**						.306	.086
** Yes**	51	63.3	54.9	53.3	48.9		
** No**	30	54.9	46.2	39.2	37.6		
**Hormone therapy**						.895	.610
** Yes**	47	58.8	42.6	49.8	40.6		
** No**	34	57.4	47.1	49.2	43.5		
**AR**						.048	.029
** AR (–)**	14	78.6	71.4	57.5	54.4		
** AR (+)**	67	53.7	40.3	47.6	39.4		
**CAG Repeat**							
** Short**	20	70.0	60.0	58.0	53.5	.050	.035
** Long**	61	49.2	39.3	43.4	38.0		

* MRM: Modified radical mastectomy.

Multivariate survival analysis was performed by testing adverse factors identified in univariate analysis in the Cox model. Only CAG repeat lengths (P = 0.004, Hazard Ratio 3.54, 95%CI 1.699–7.381) and lymph node status (P = 0.001, Hazard Ratio 3.84, 95%CI 1.589–9.317) retained prognostic significance for 5y-OS. AR expression (P = 0.016, Hazard Ratio 2.55, 95%CI 1.113–5.843) and lymph node status (P = 0.001, Hazard Ratio 3.65, 95%CI 1.742–7.567) had prognostic significance for 5y-DFS.

## Discussion

MBC remained an extremely rare disease. Although less frequent, it tended to be more aggressive than its female counterpart [7]. Most of our current knowledge regarding its biology, natural history and treatment strategies had been extrapolated from its female counterpart. Estrogen and progesterone were thought to play an important role in development and progression of breast cancer in women. However, the role of androgen in breast cancer etiology was poorly understood. Wang [8] found better survival in ER-positive MBC patients but did not find a benefit from treatment with Tamoxifen. More recently, Pich [9] found no correlation between the expression of ER or PR with overall survival. They concluded that MBC patients are biologically different from female breast carcinoma (FBC) patients and questioned the rationale for the use of anti-hormonal (Tamoxifen) therapy in MBC patients. Much research was needed to characterize further the molecular biological properties of MBC and their prognostic significance, and to devise treatment strategies, including optimal endocrine regimens. In our study, AR-positive expression was detected in 82.7% of MBC patients; this rate was identical with that reported (81%) by Noman Kidwai [10] and similar to the rate of 74% observed by Pich [9]. Moreover, AR protein expression was related to ER and PR protein expression in our study. Analogous other studies on FBCs or MBCs, we found a correlation between AR and ER or PR status [7], [11]. This correlation mechanism will require us to continue to study in MBC.

The role of AR expression as a prognostic factor was controversial. In MBC, Pich [9] showed lack of association between AR and survival, whereas Munoz [12] suggested that decreased androgen action (AR-negative) within the breast might contribute to an earlier development of MBC. In contrast, we found a strong correlation between AR protein expression and survival (5y-OS and 5y-DFS) in MBC patients. AR-positivity was associated with adverse prognosis and AR status had prognostic significance in univariate analysis. The similar findings [7] together with our results might indicate a new opinion of endocrine therapy (anti androgenic therapy) for MBC patients.

The involvement of AR in MBC development had been also investigated at the DNA level [5], [6], [13]. AR activity could be affected by the highly variable polyglutamine tract (CAG repeat) located in the NH2-terminal trans activation domain of the AR. Expansion of the CAG repeat had been associated with reduced AR expression/transactivation, whereas the relatively short CAG repeat sequence increased the level of transactivation of the AR [14]. Interestingly, in our study long CAG repeat sequence was associated with increased AR expression appeared in the tumor tissue and the rate was 90%. This phenomenon was worthy of our further research. Furthermore, we should continue to explore and discover the regulatory factor of the nucleic acid to protein transcription and inhibit this regulatory factor which might inhibit tumor development and metastasis.

We initiated to test CAG repeat length of blood and tumor tissue in the same MBC patient. Interestingly, the test results of two paired samples were the same in each patient. As the incidence of MBC was low, the size of our study cohort was small. Although there were only 31 MBC patients who had paired samples, these consistent results supported two paired samples information substituted for one another. We had observed statistically significant difference between the CAG repeat length of MBC patients and controls. In addition, no males in the control group had alleles containing more than 28 CAG repeats. Only one of the MBC patients had an allele containing 14 repeats, compared to 6 of the controls. The length of this CAG repeat had been investigated in several studies of MBC patients and controls previously [5], [6], [15]. Our study was consistent with the Australian study [15] that found statistically significant difference in CAG repeat length between MBC patients and controls. To our knowledge, there were no data on prognostic value of AR (CAG)n repeats status in MBC. However, it was found to be significantly different survival between long and short CAG repeat sequence in MBC patients. We believed that a relatively long CAG repeat sequence within the AR gene might be implicated in a few cases of MBC and poor prognosis. Conversely, a short CAG repeat sequence might offer a degree of protection against male breast cancer.

This study revealed the following important findings: (a) there was a significant difference in the CAG repeat lengths between MBC patients and controls; (b) long CAG repeat lengths were more common in patients than controls and there was a strong trend toward short CAG repeat lengths being more prevalent in controls than in cases; (c) long CAG repeat sequence presented at the T2-4 stage compared with short CAG repeat sequence in MBC patients but did not differ with respect to other clinicopathological features; (d) there was a significant difference by ER and PR status regarding AR expression in the MBC patients; (e) the presence of long CAG repeat sequence and AR-positive expression were associated with shorter survival of MBC patients.

To conclude, the findings presented in this study indicate that the CAG repeat length within the AR gene might be one useful molecular biomarker to identify males at increased risk of breast cancer development. The CAG repeat length and AR expression were two independent prognostic indicators in MBC patients. Larger studies were required to define the importance of AR (CAG)n repeats status and AR expression in MBC further and we had agreed to contribute our data to this.

## Supporting Information

Figure S1 We detected CAG repeat length of bloods and tumor tissues for 31 MBC patients. The test results of two paired samples were the same in each patient.
(DOC)
