# Supplementary material for: Long CAG Repeat Sequence and Protein Expression of Androgen Receptor Considered as Prognostic Indicators in Male Breast Carcinoma
Source: PLoS One. 2012 Dec 14;7(12):e52271. doi: 10.1371/journal.pone.0052271 (PMC3522691; doi:10.1371/journal.pone.0052271)

Supporting Information

We detected CAG repeat length of bloods and tumor tissues for 31 MBC patients. The test results of two paired samples were the same in each patient.

The test results are as follows:

No.1


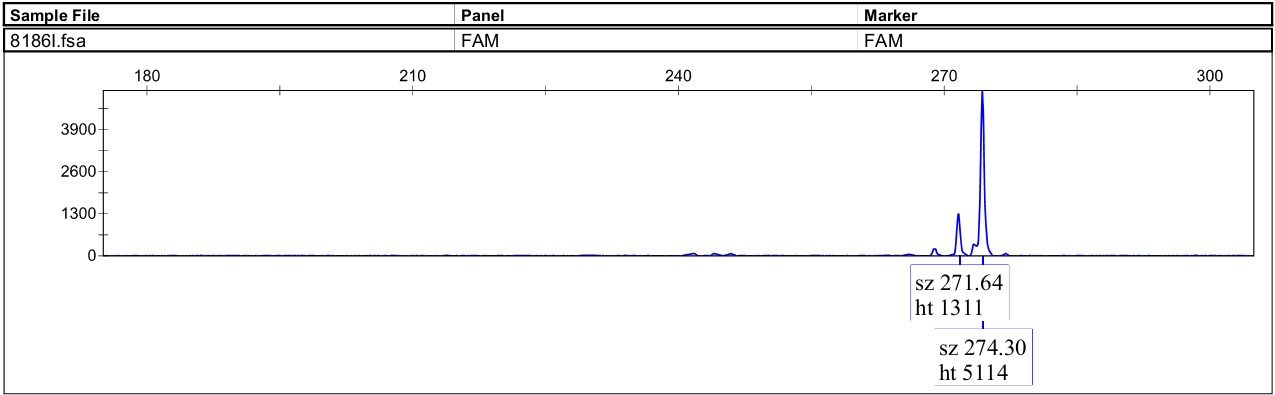


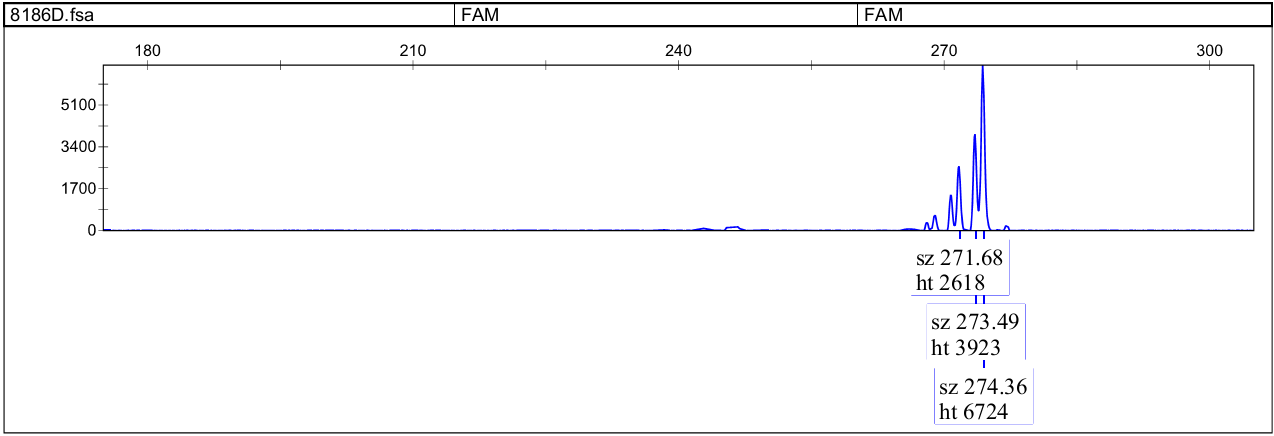


No.2


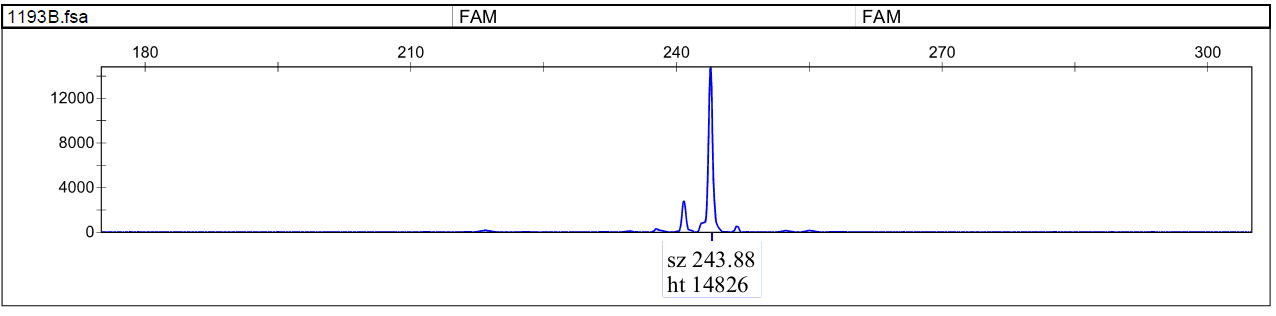


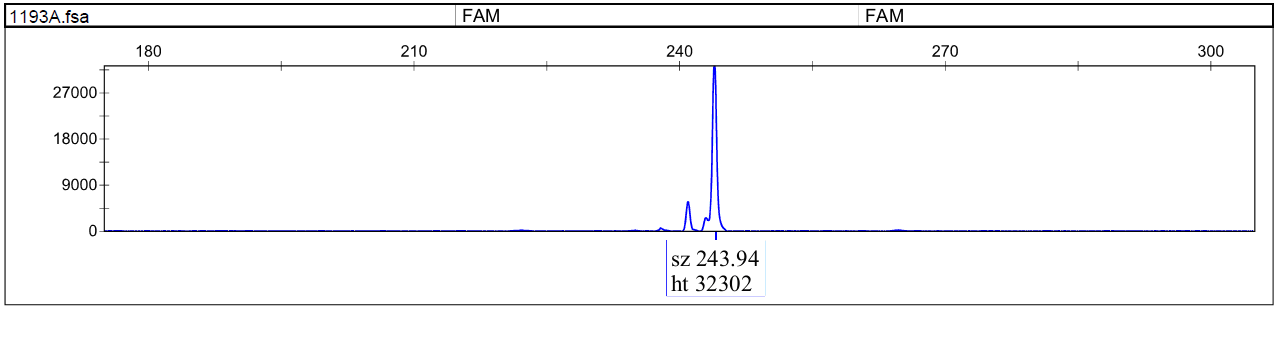


No.3

**
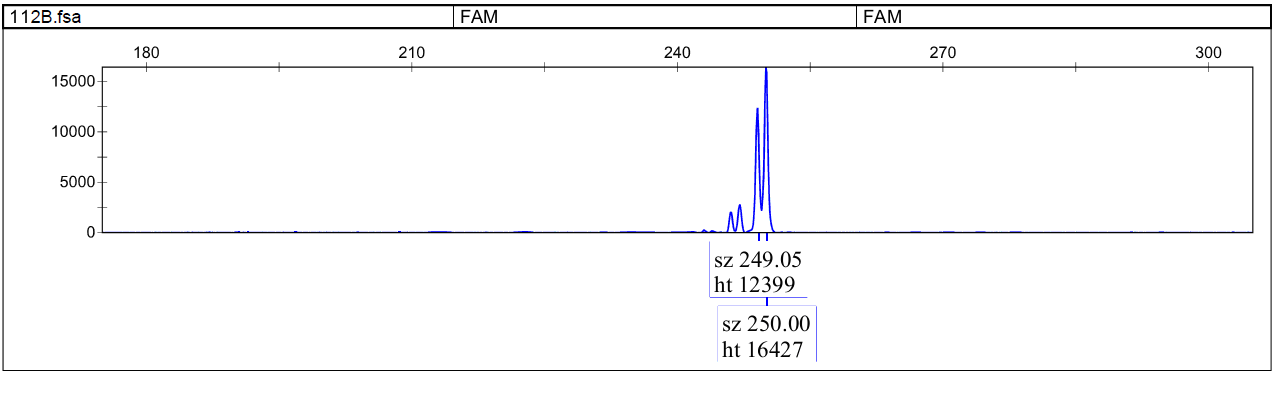
**

**
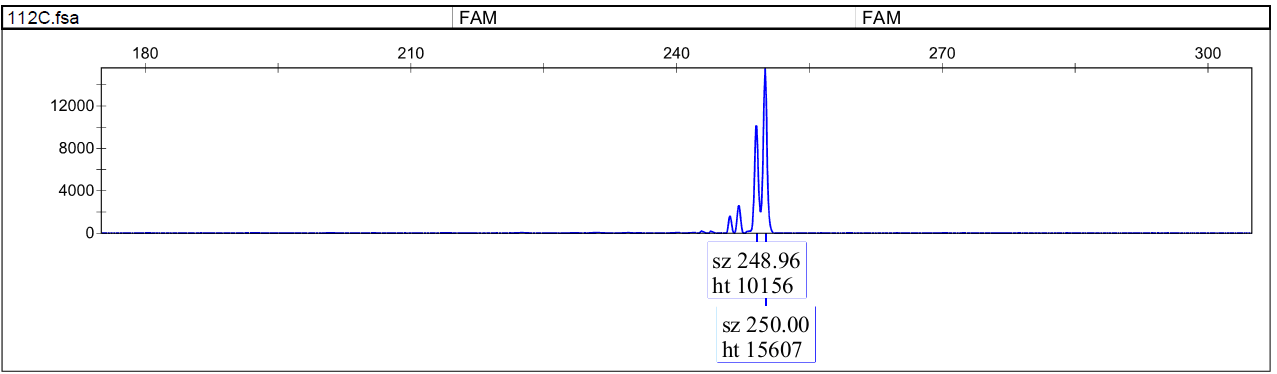
**

No.**4**


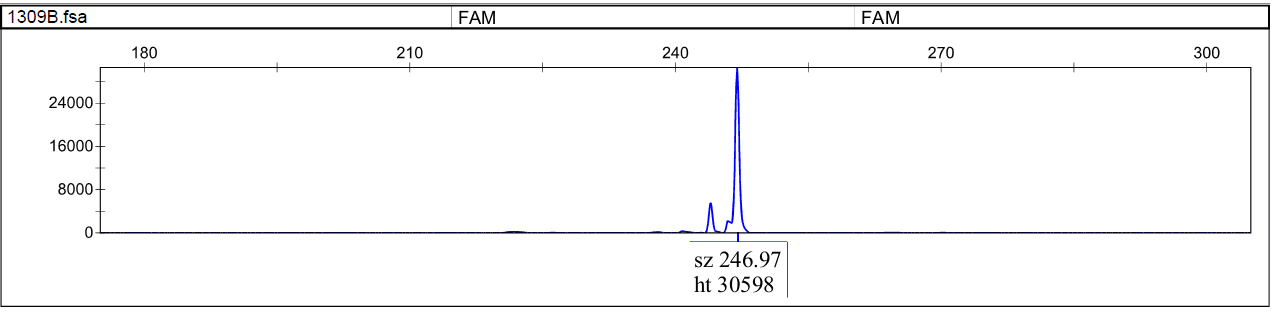


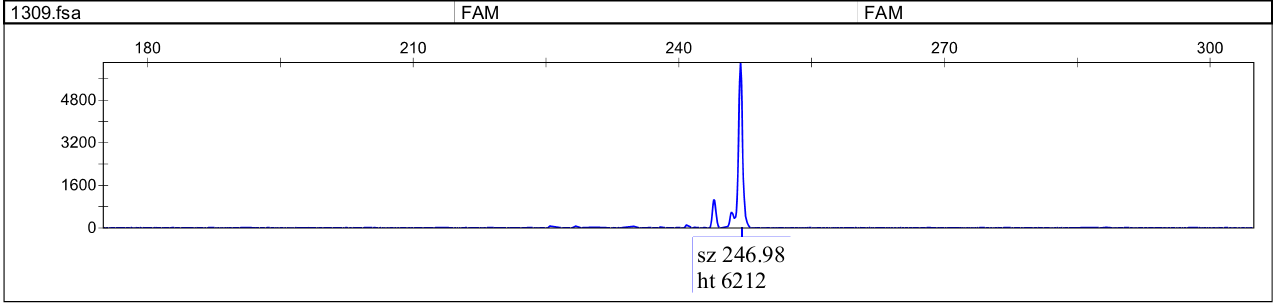


No.5


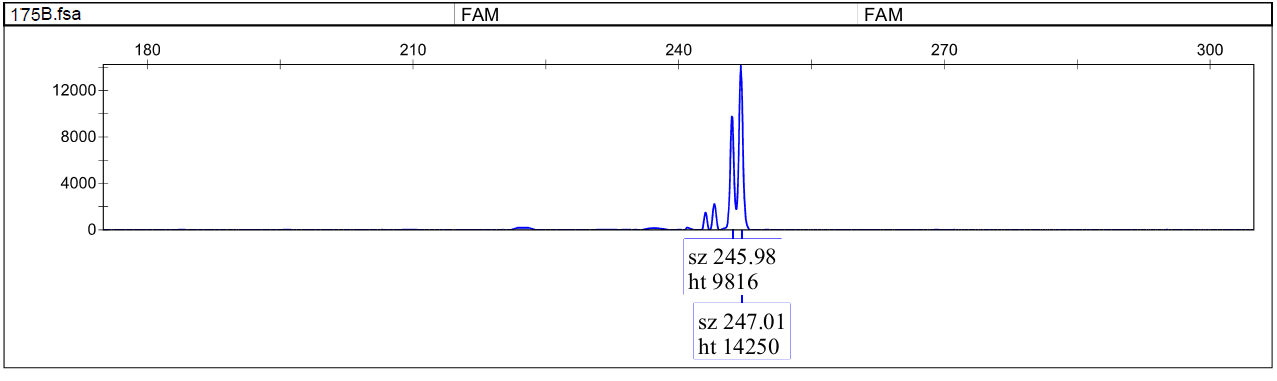


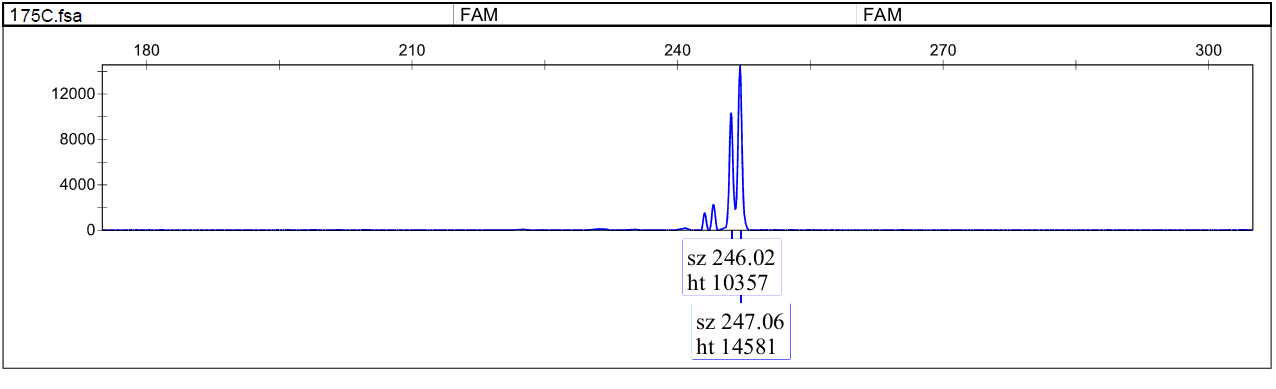


No.6


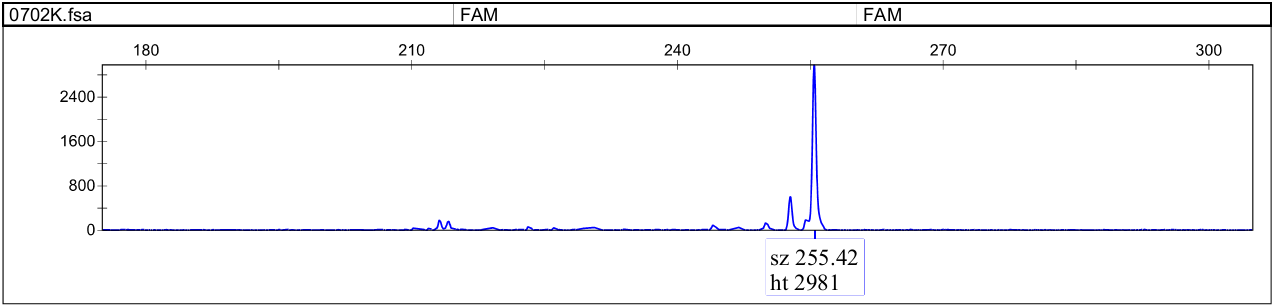


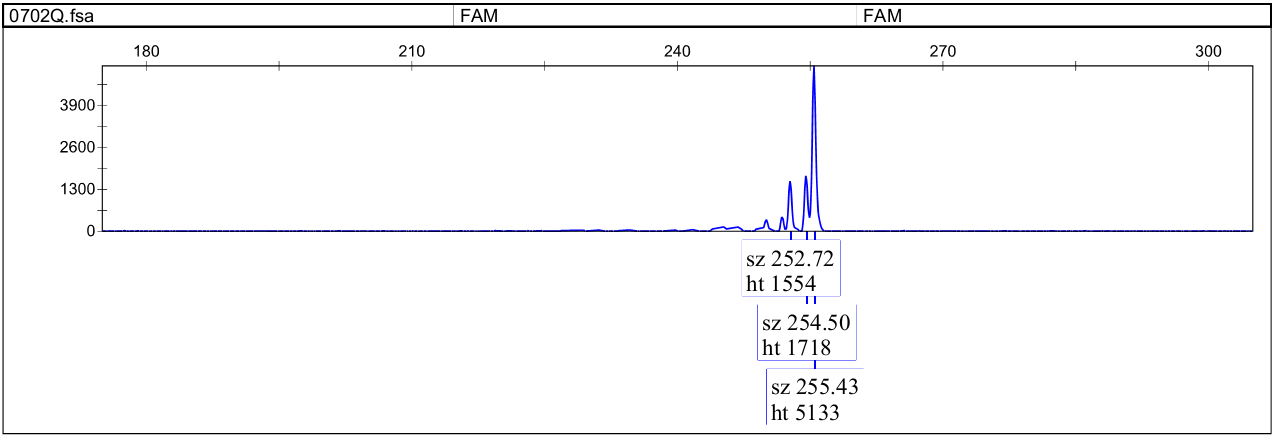


No.7


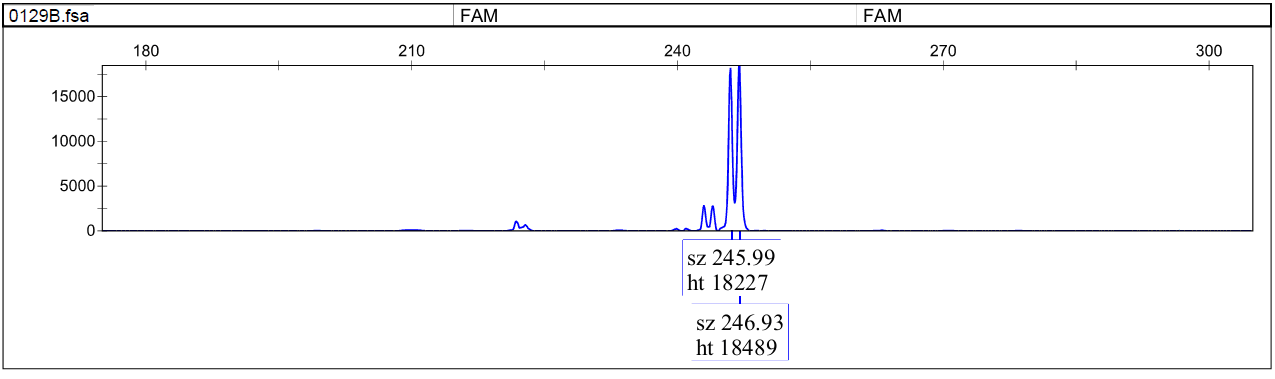


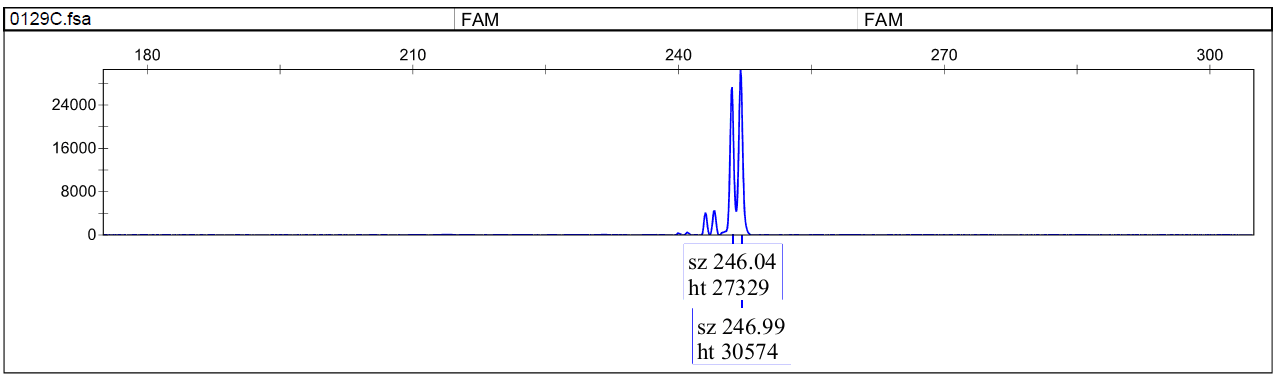


No.8


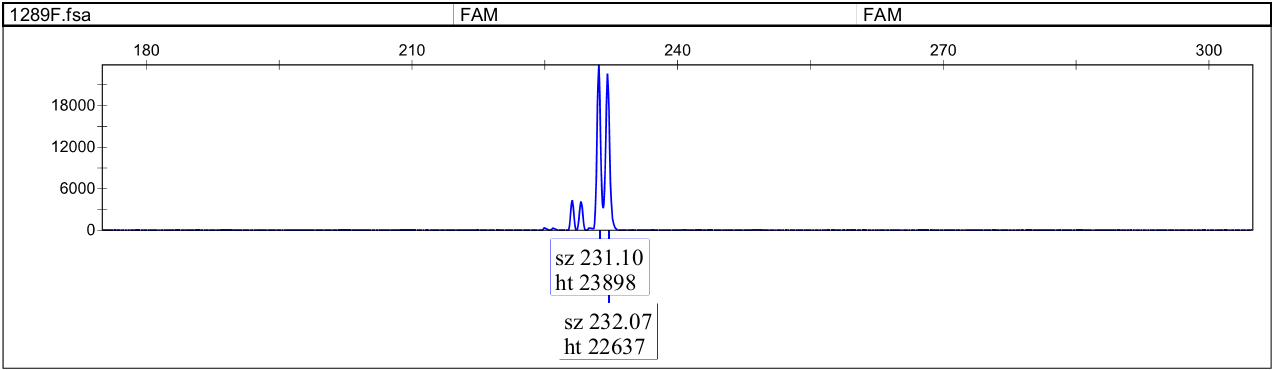


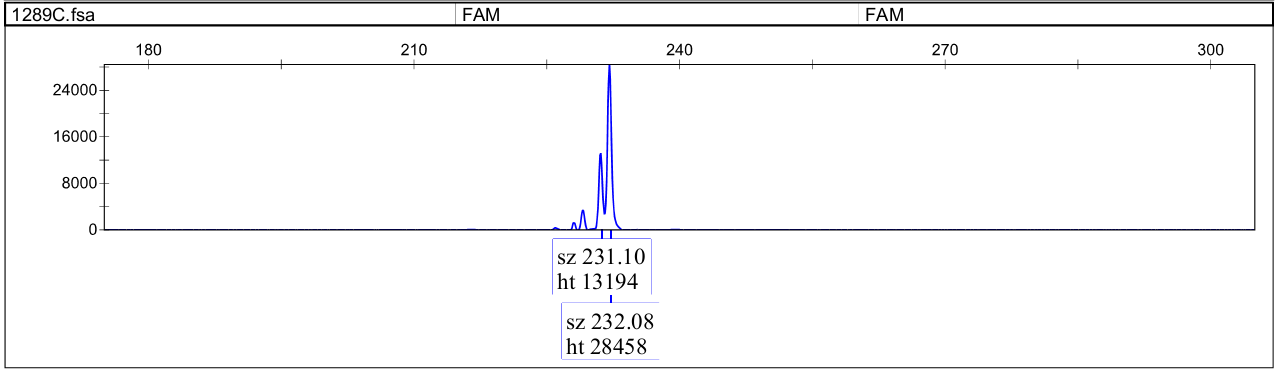


No.9


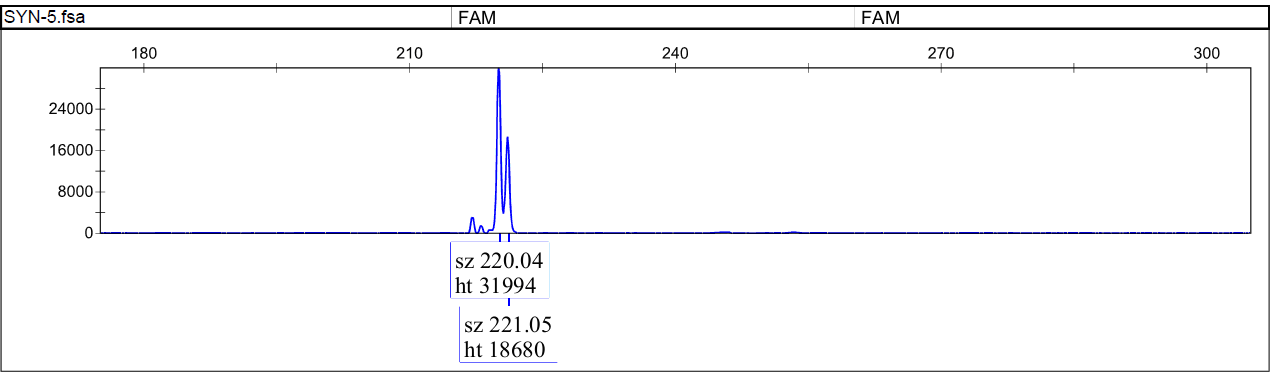


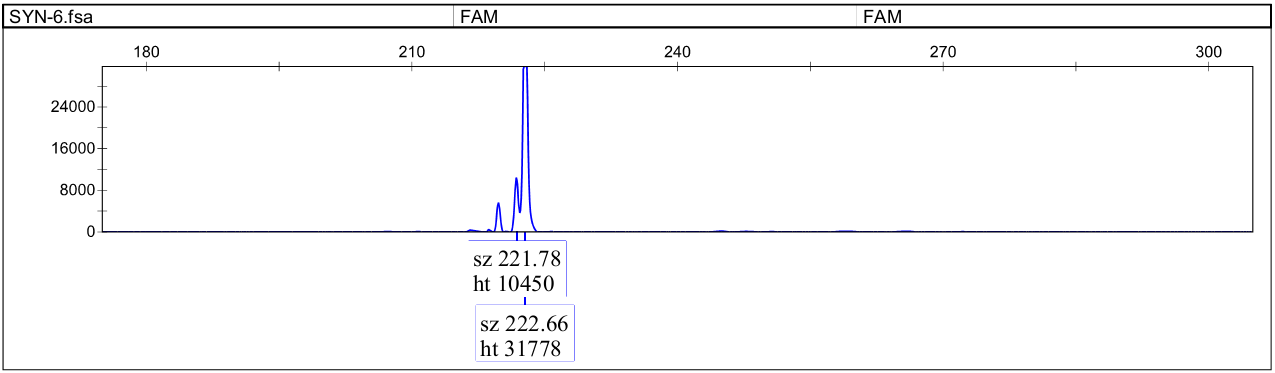


No.10


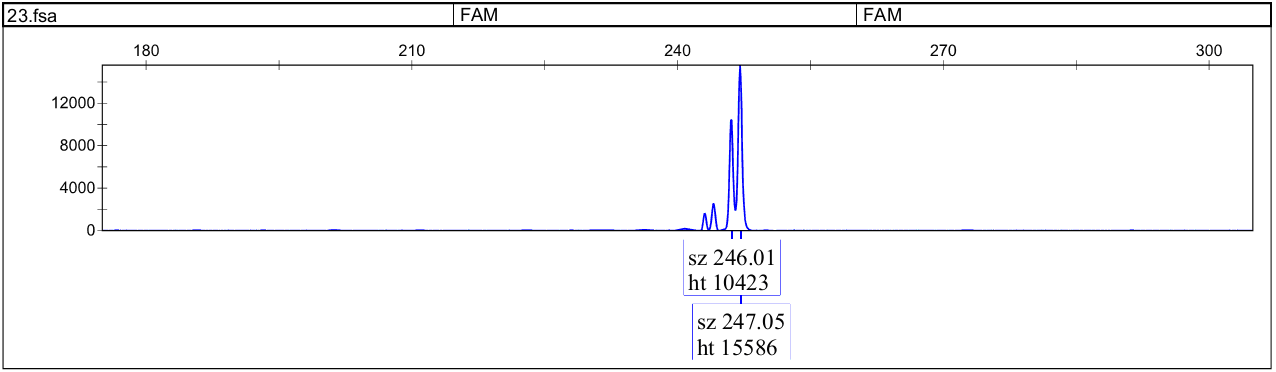


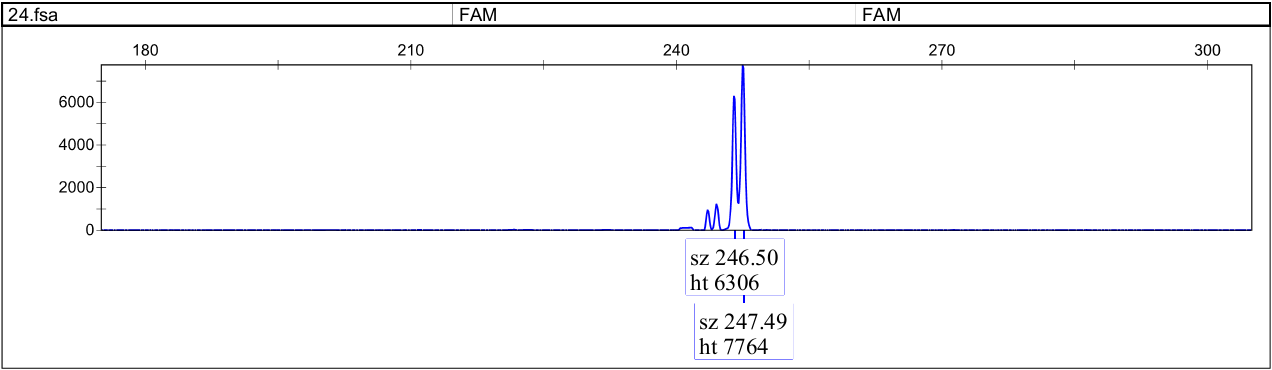


No.11


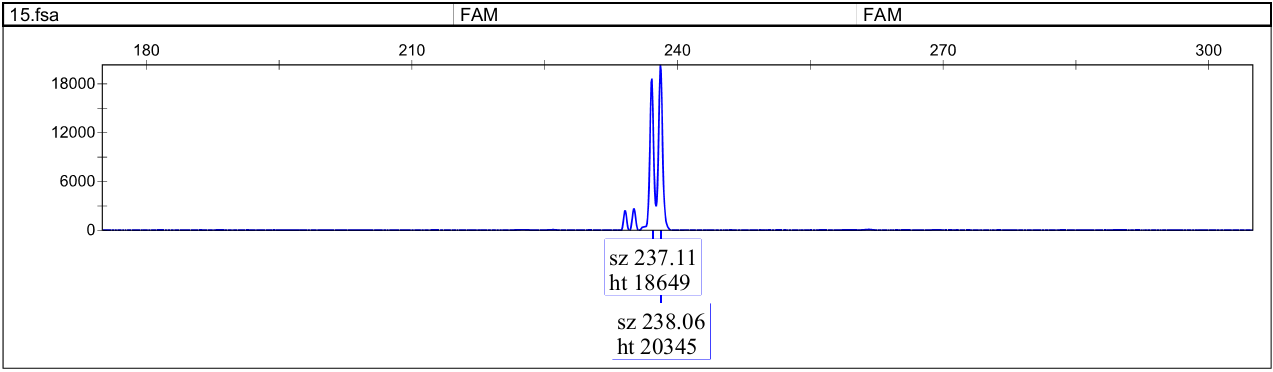


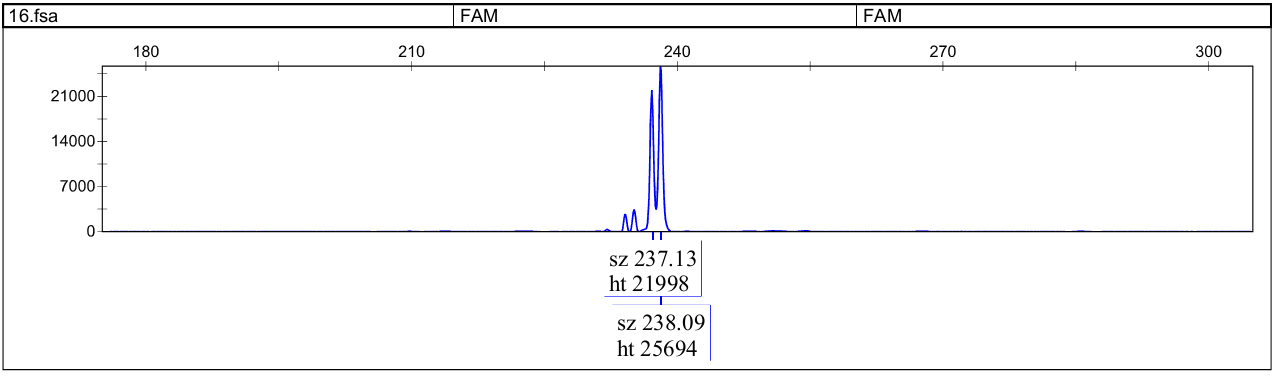


No.12


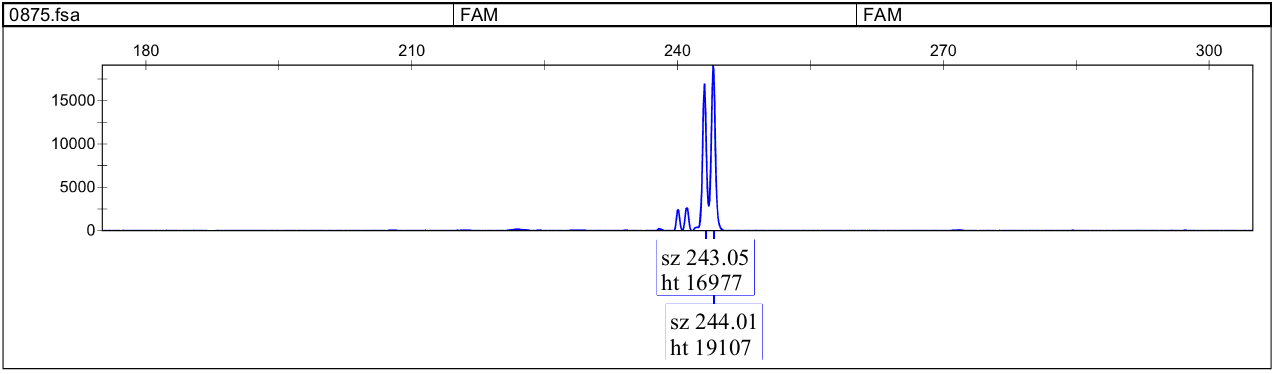


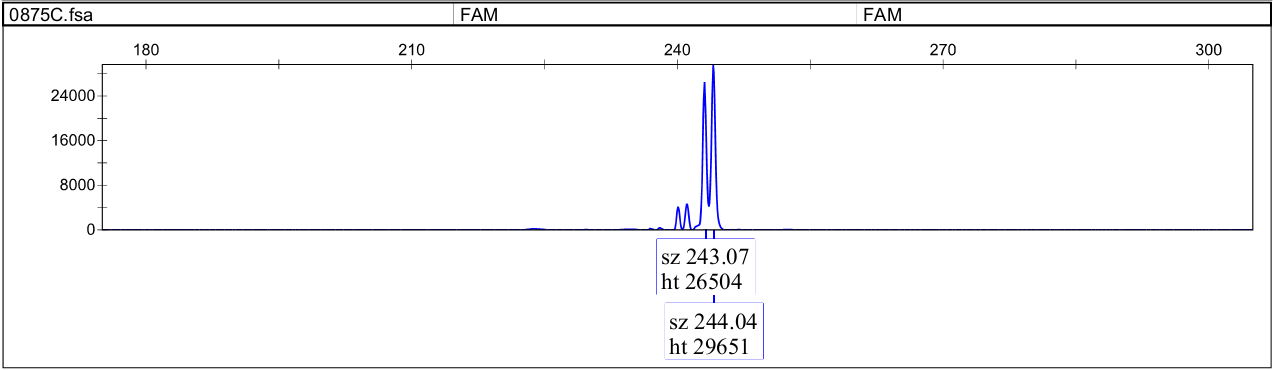


No.13


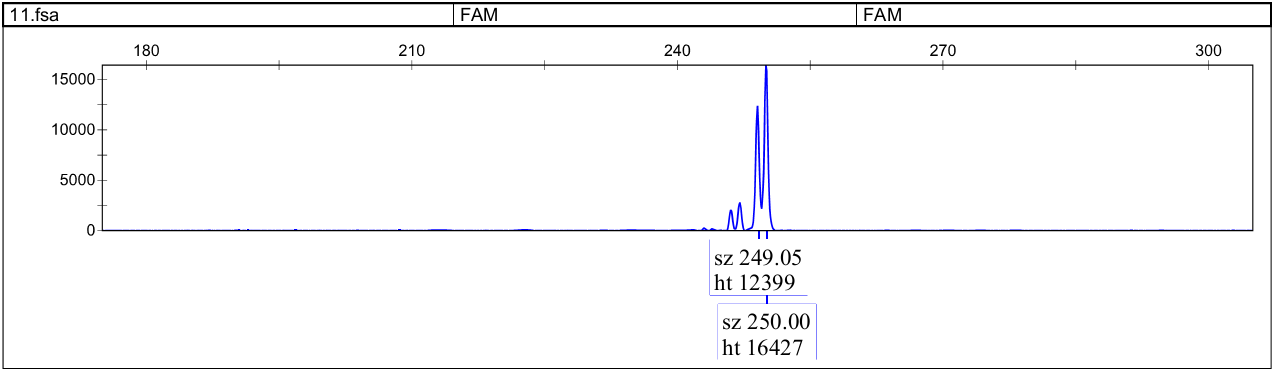


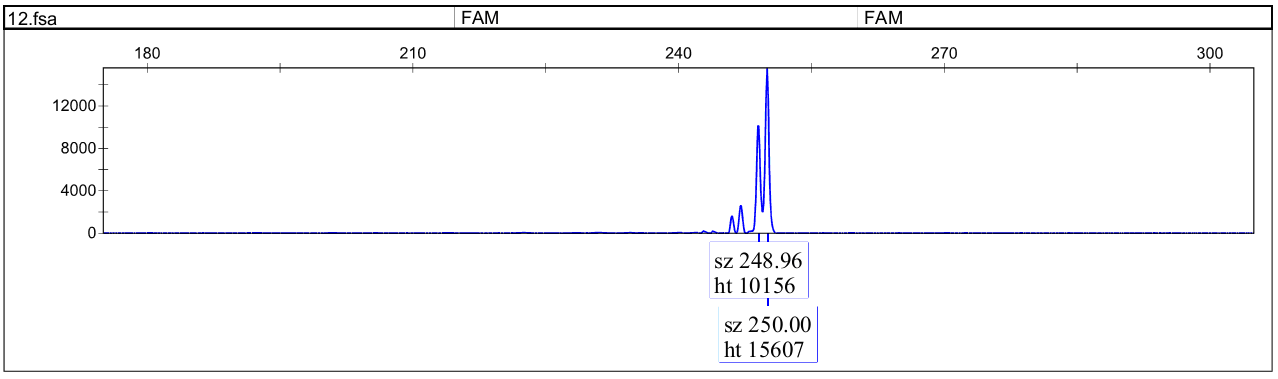


No.14


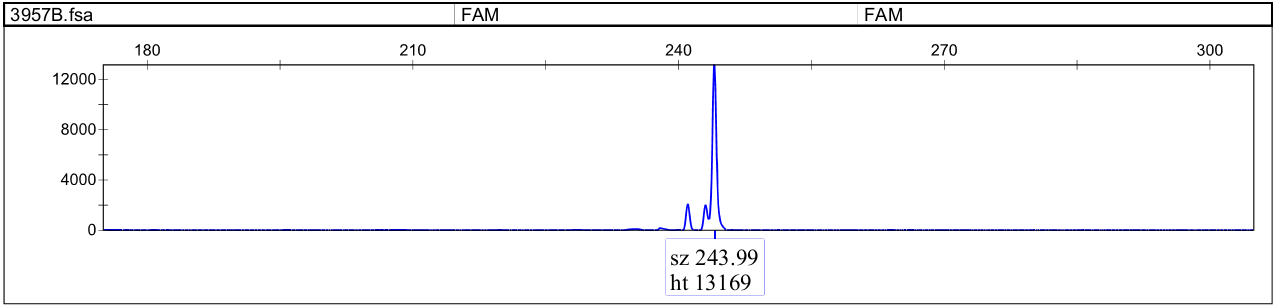


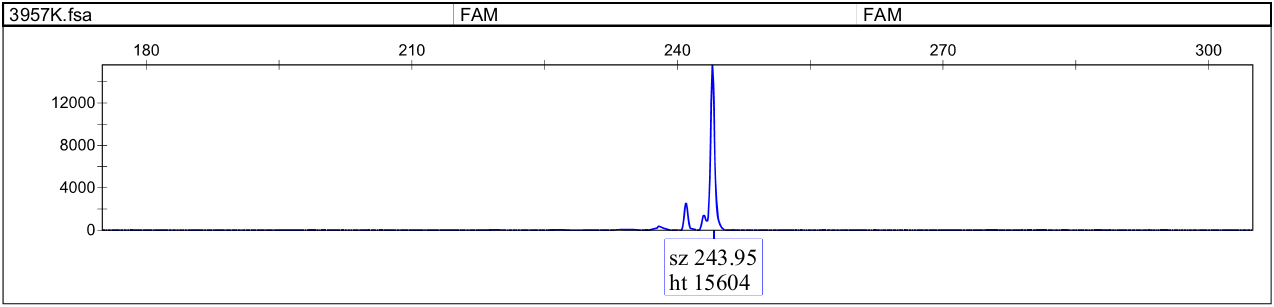


No.15


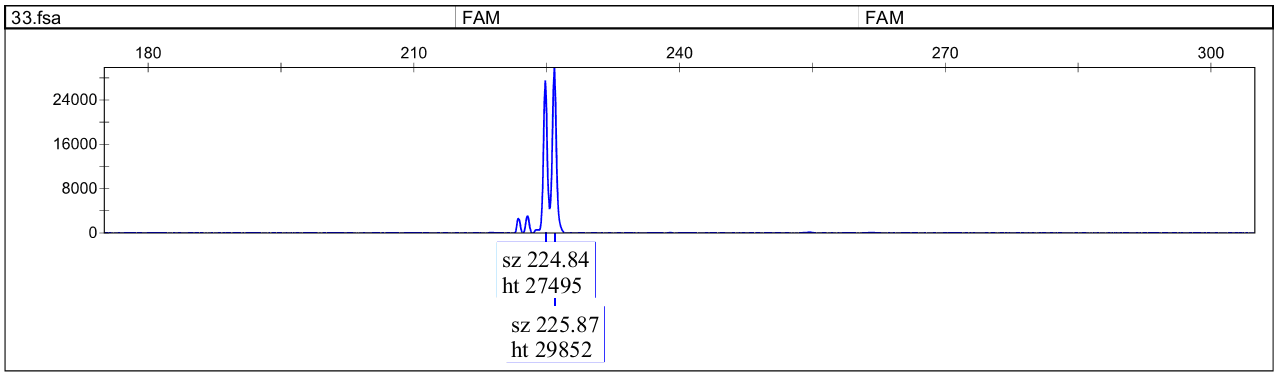


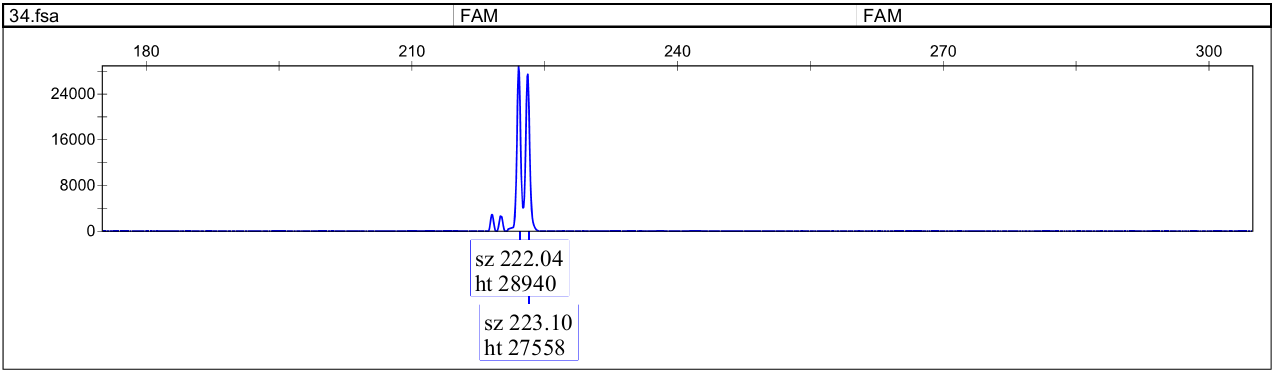


No.16


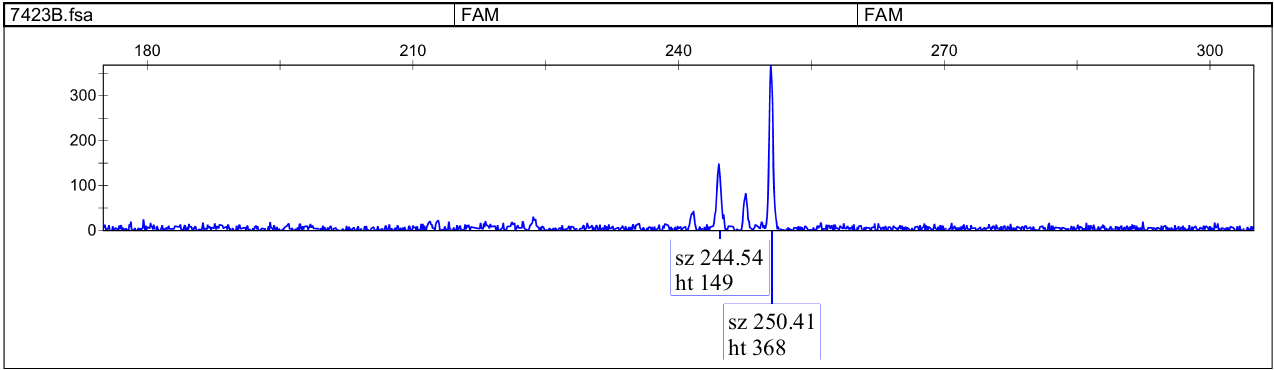


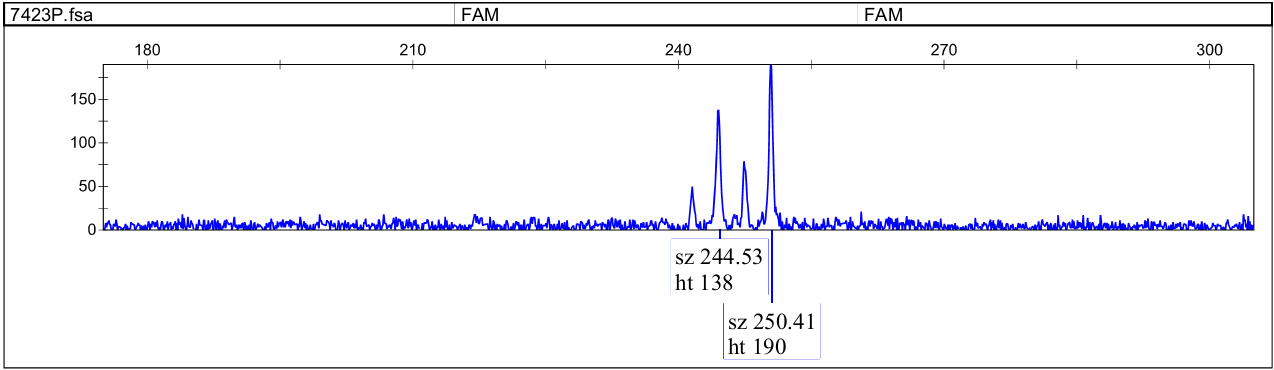


No.17


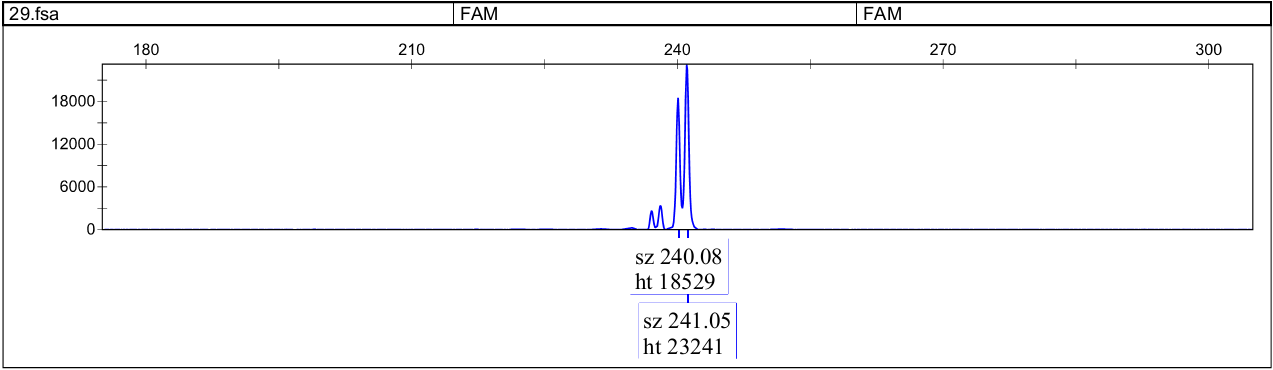


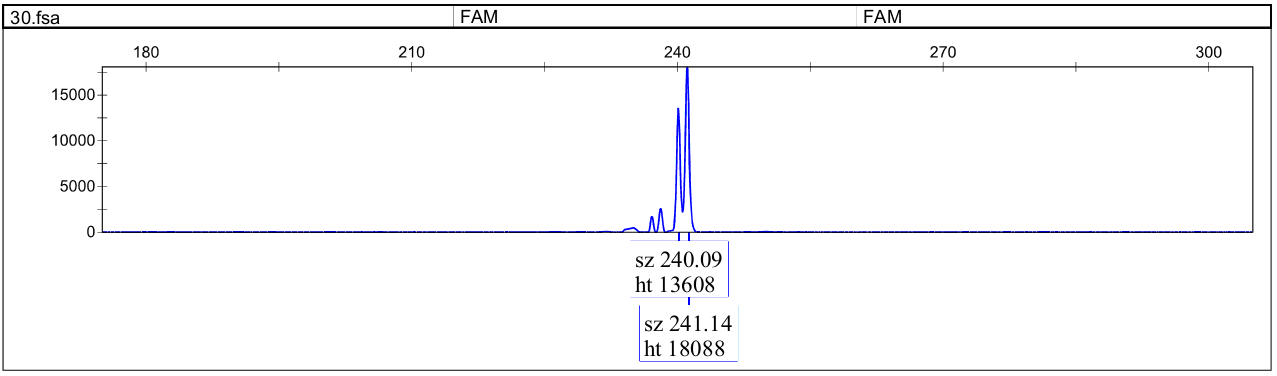


No.18


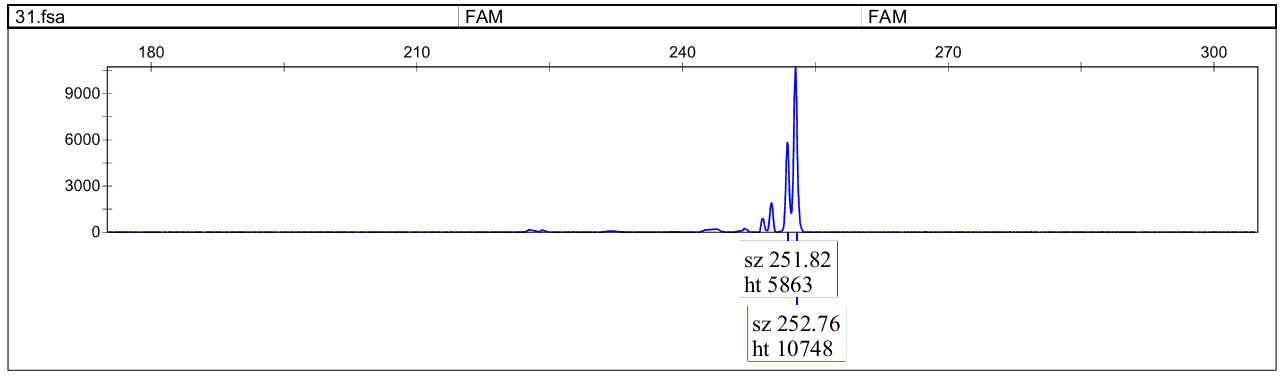


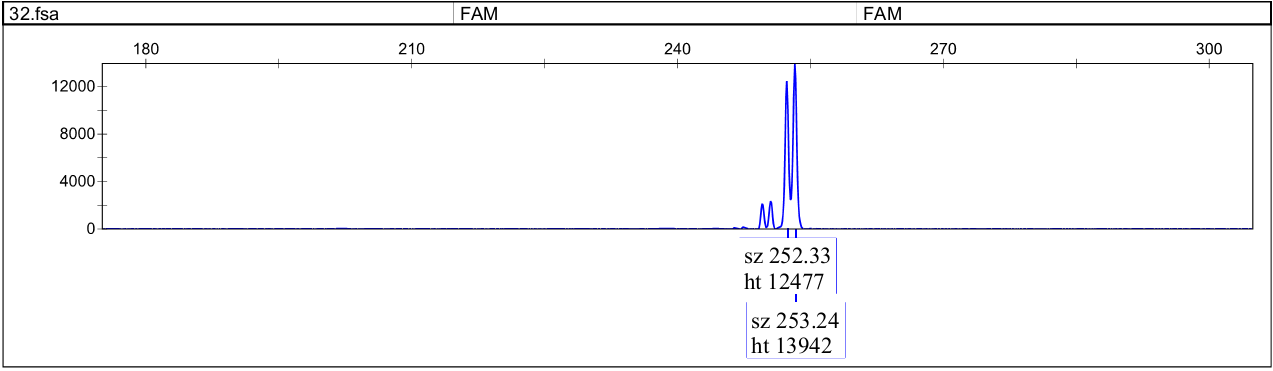


No.19


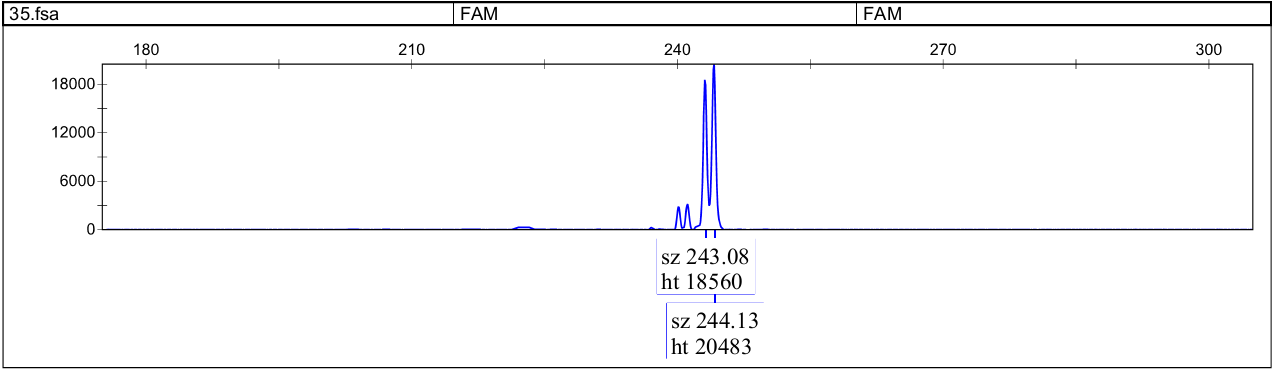


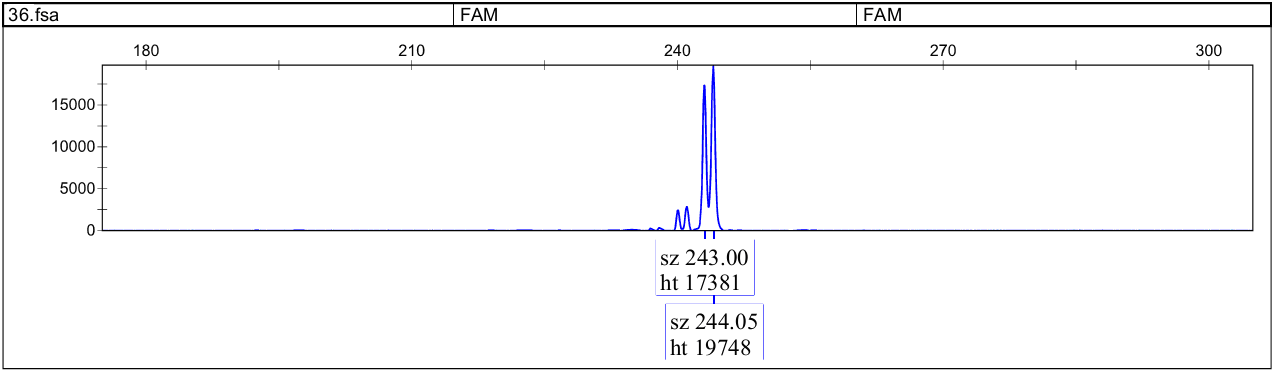


No.20


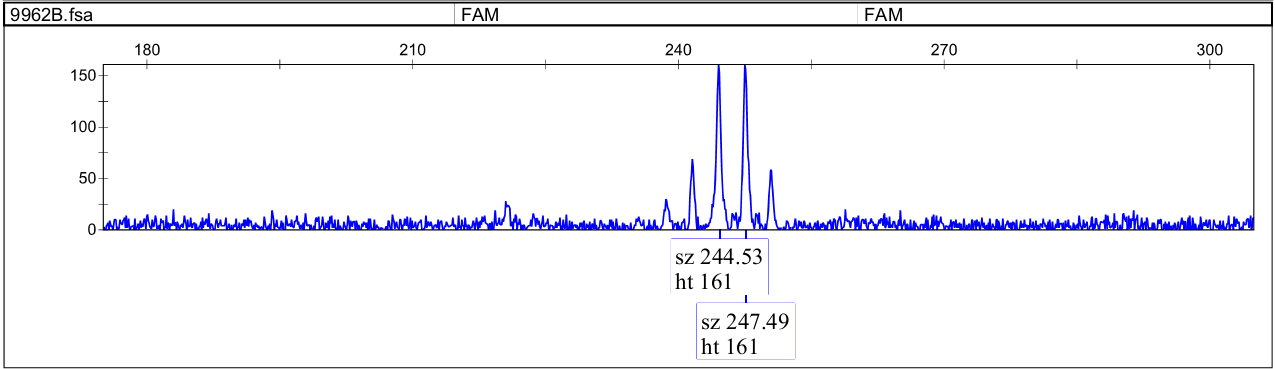


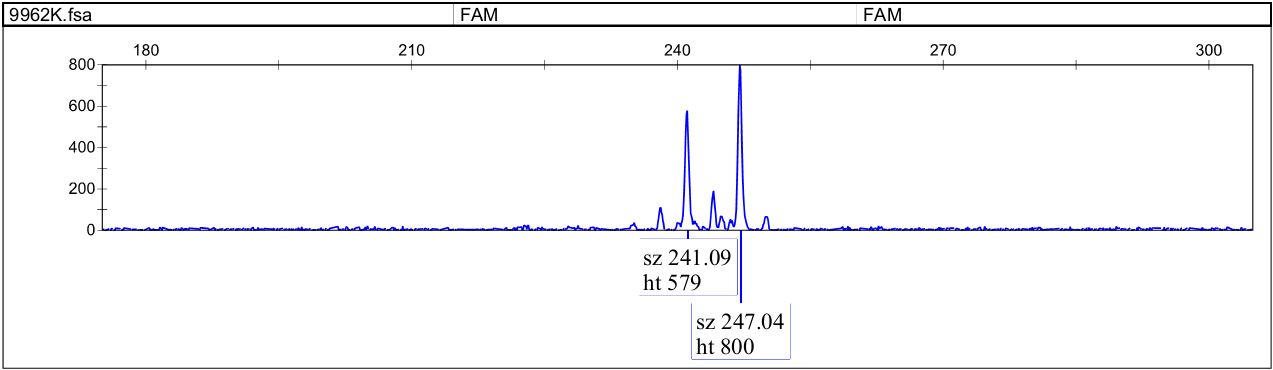


No.21


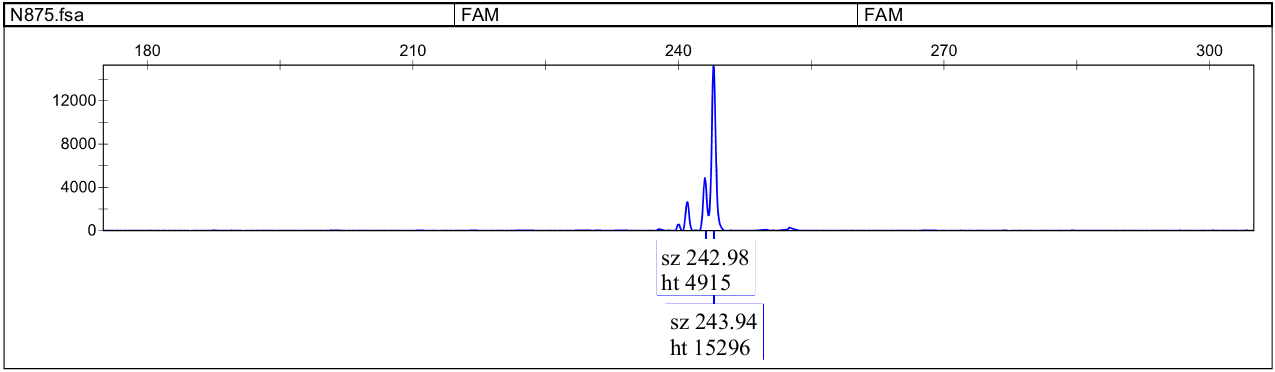


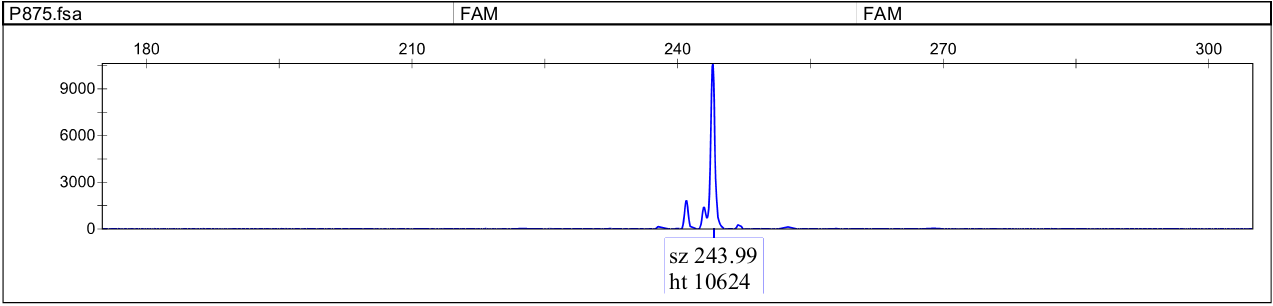


No.22


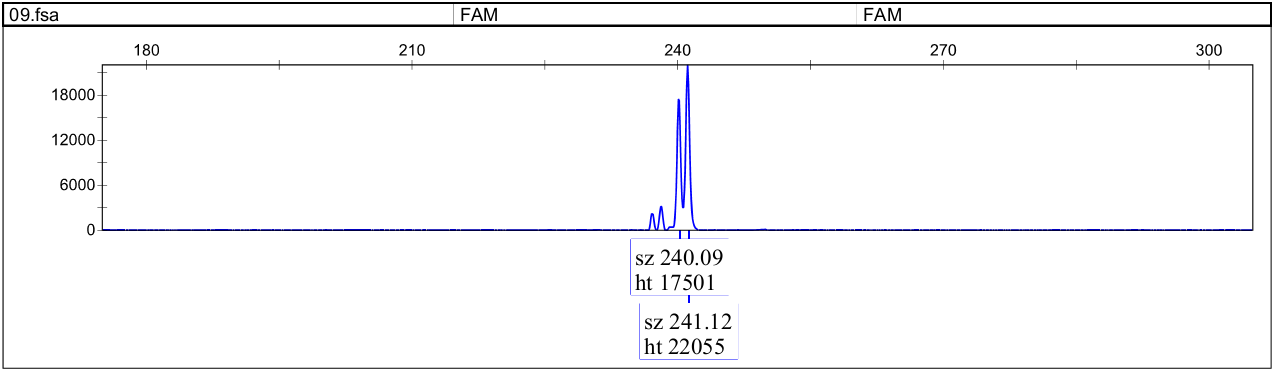


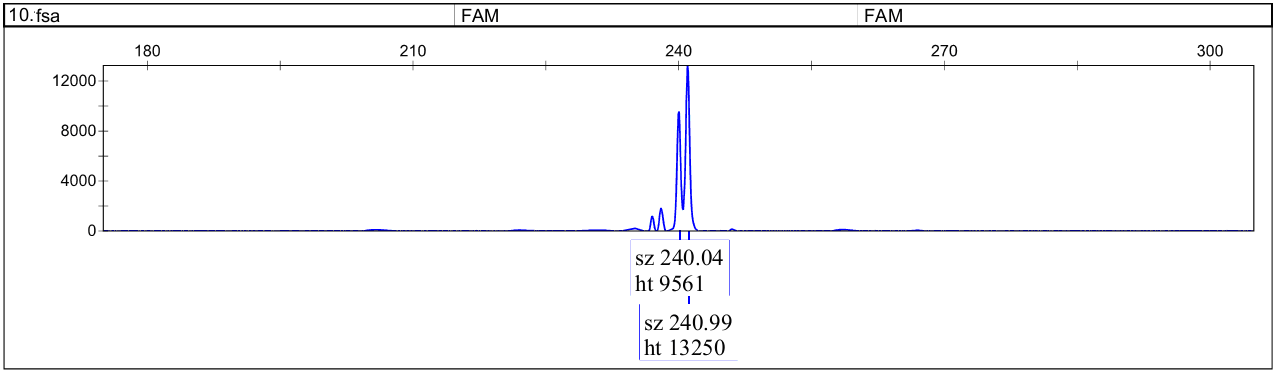


No.23


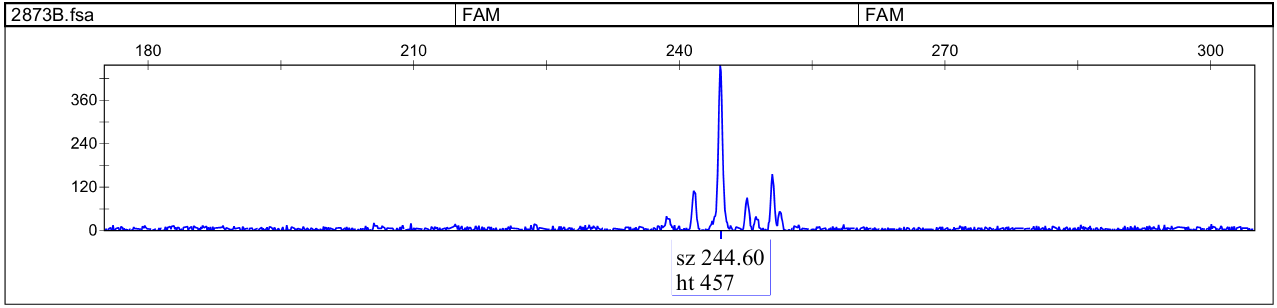


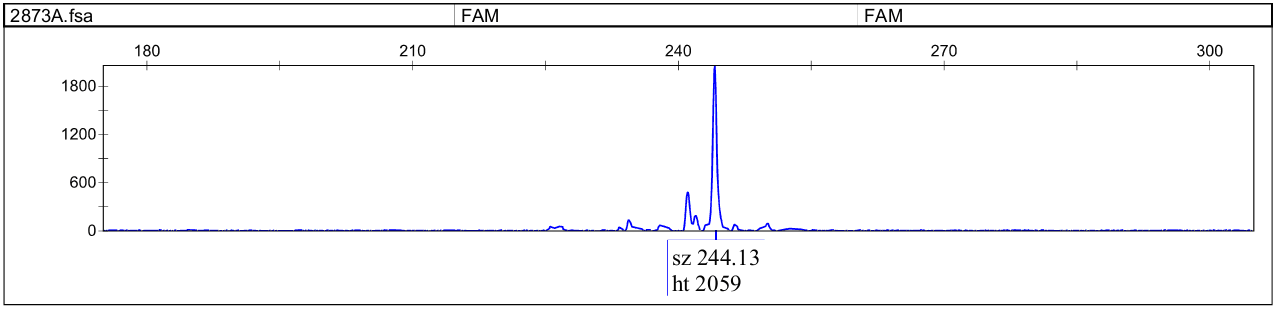


No.24


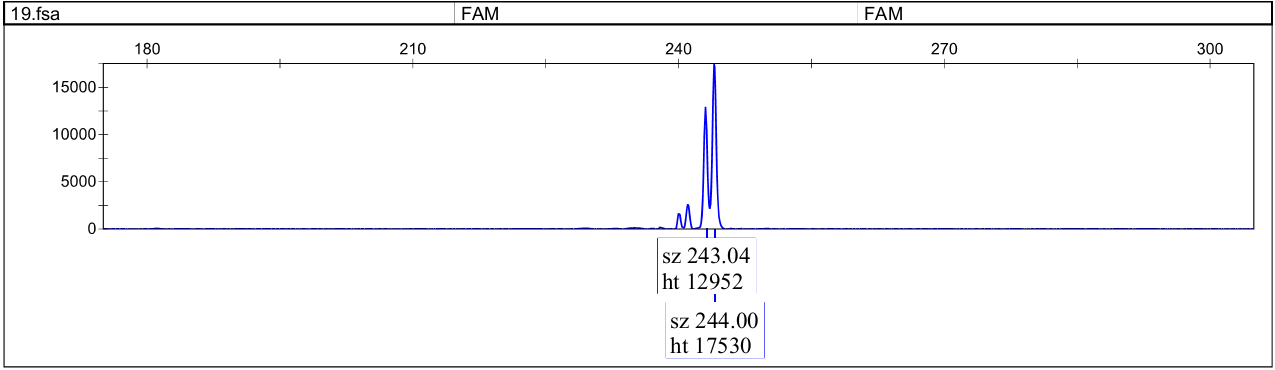


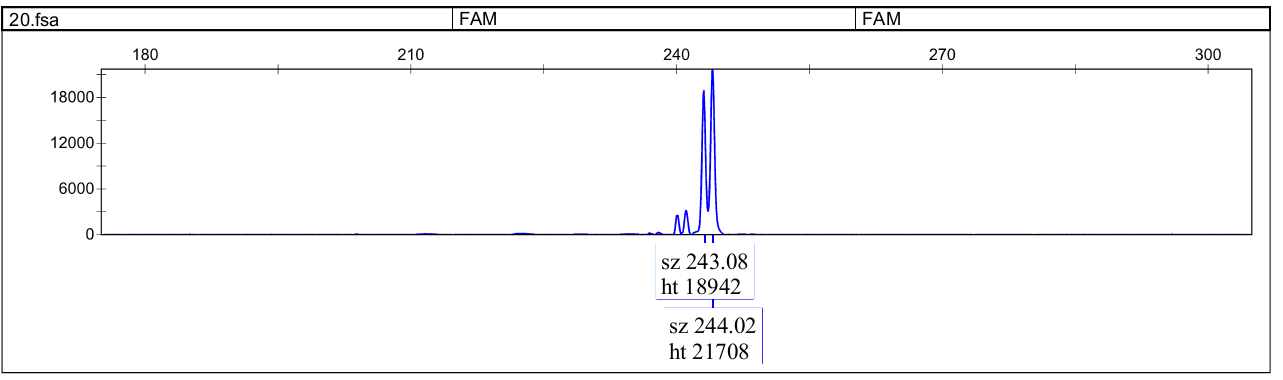


No.25


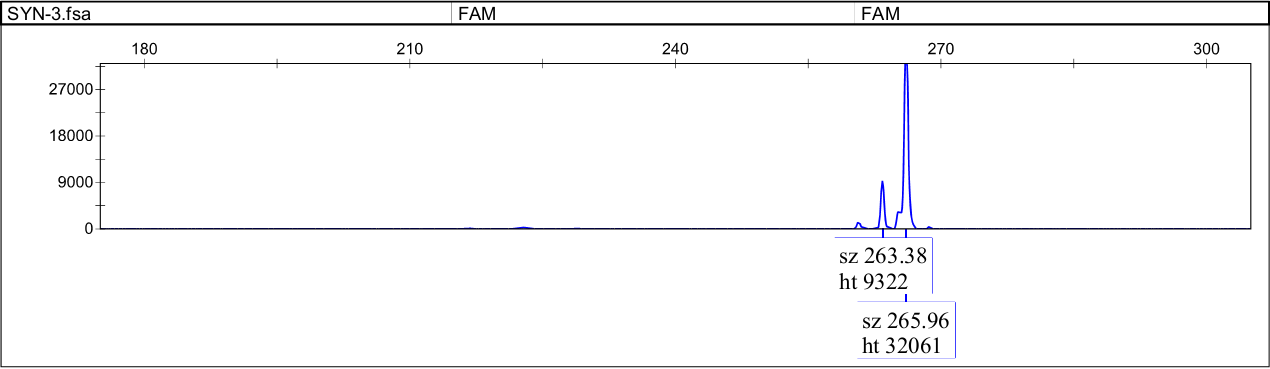


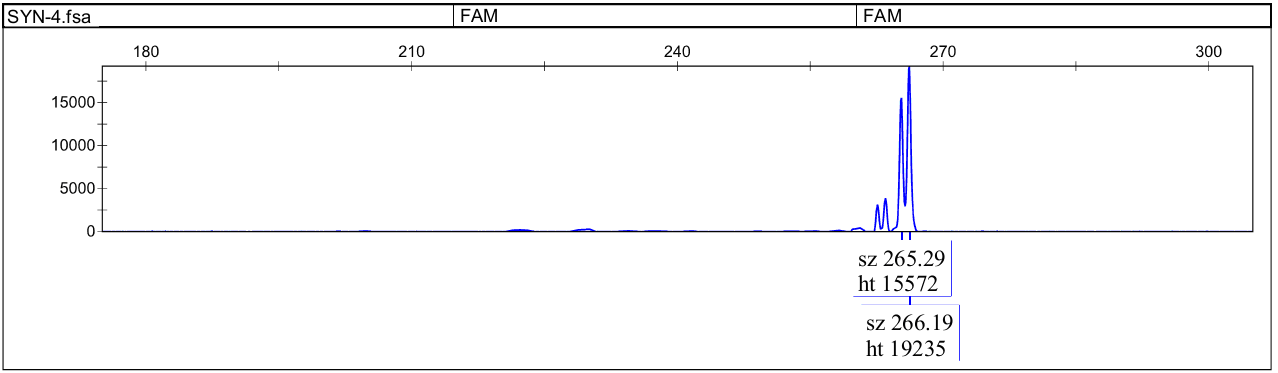


No.26


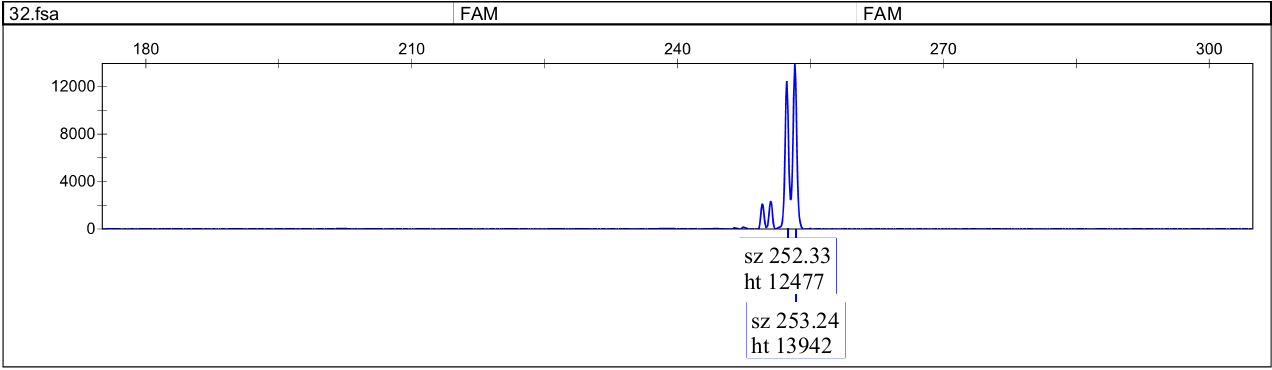


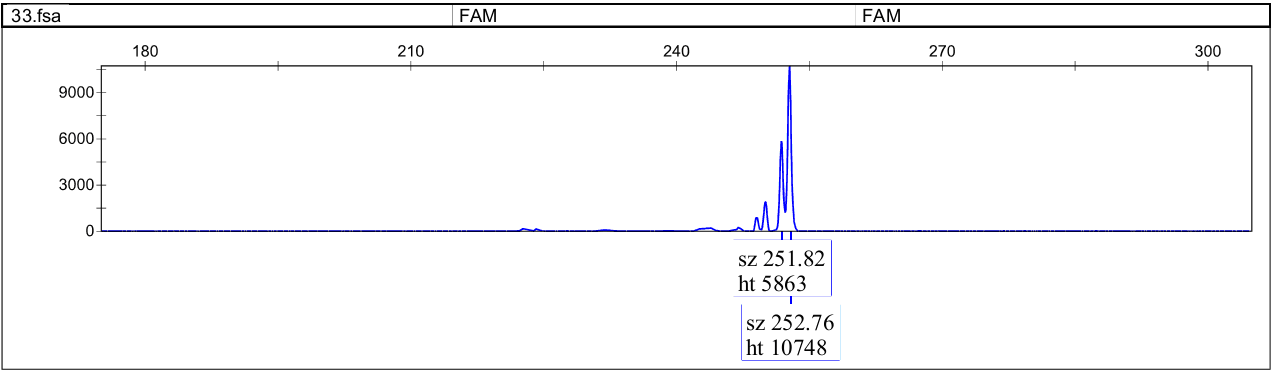


No.27


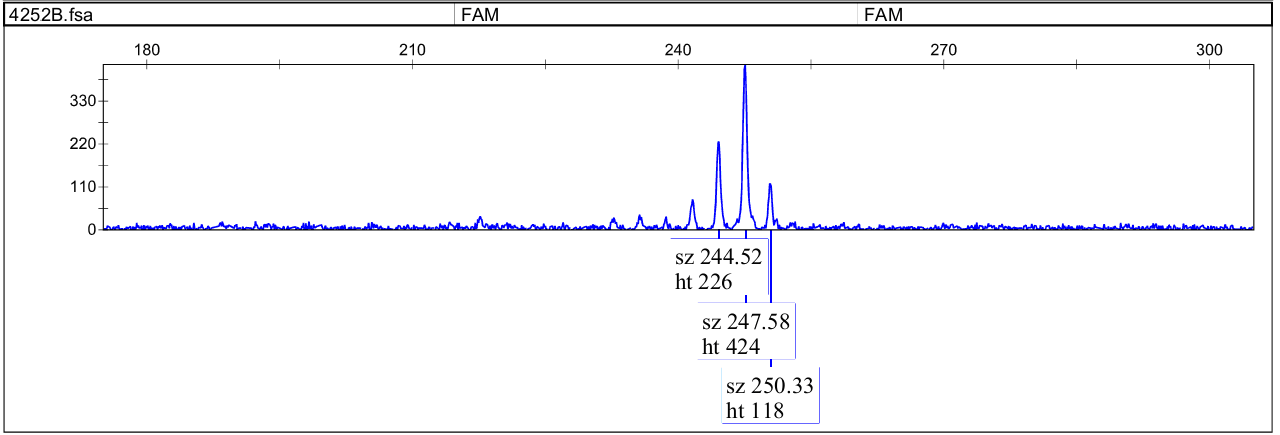


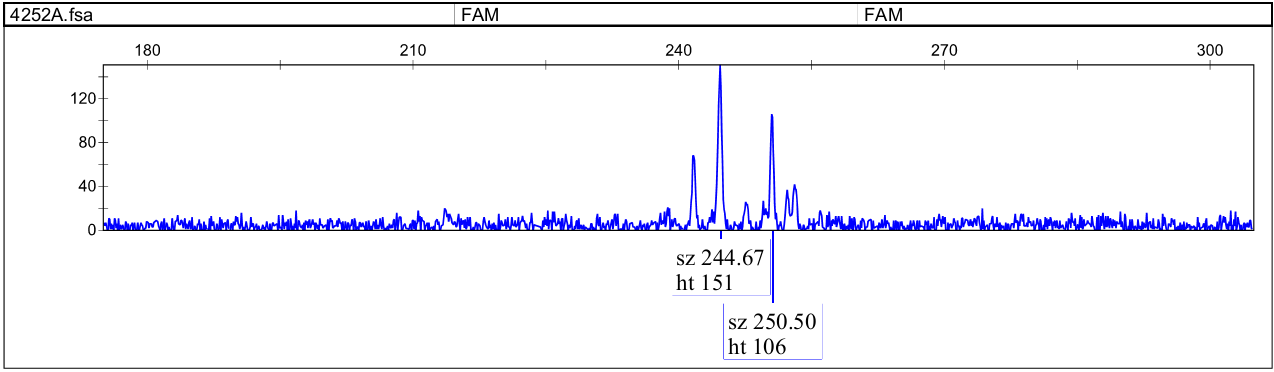


No.28


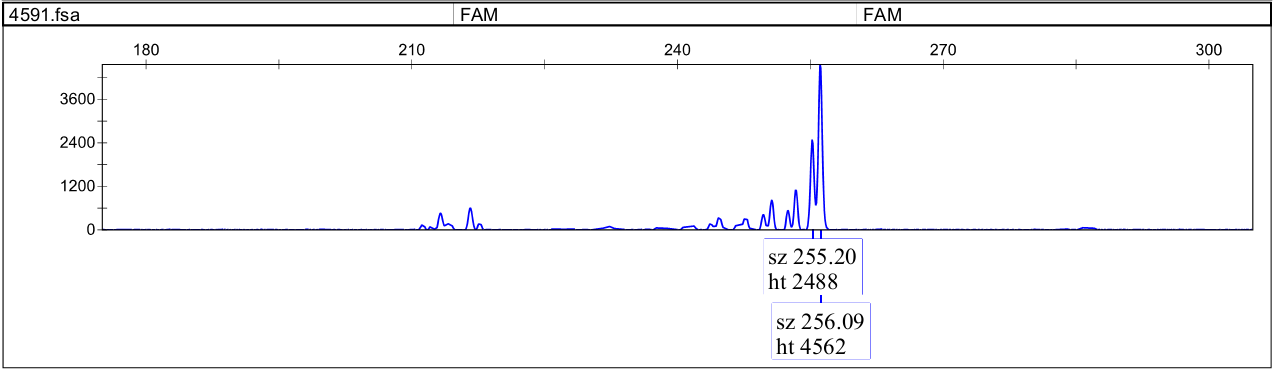


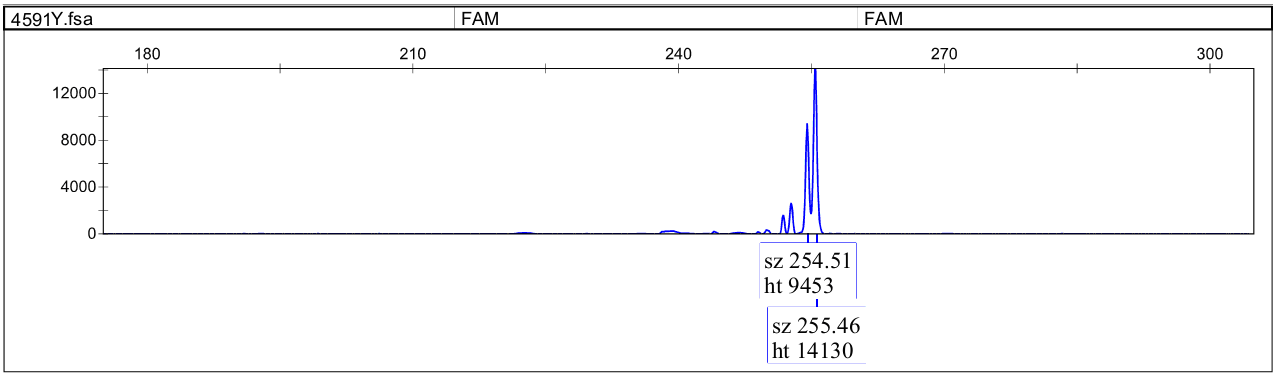


No.29


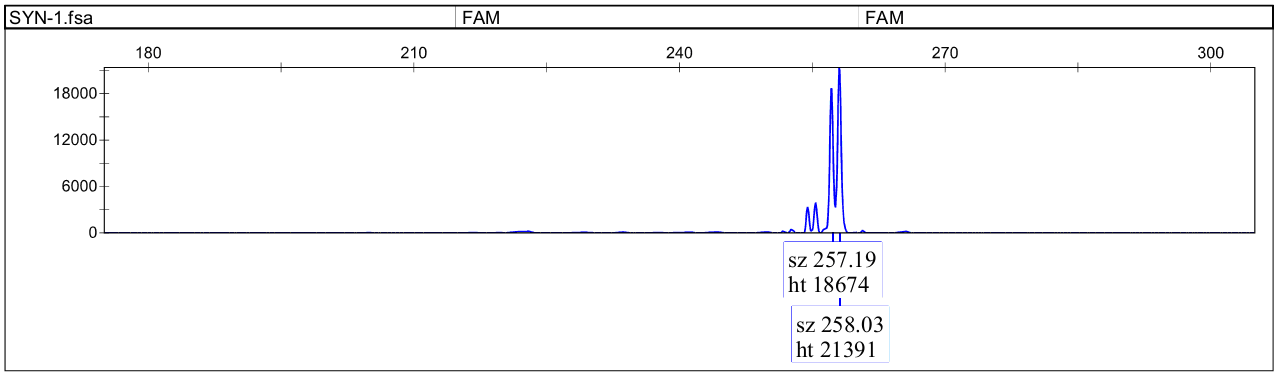


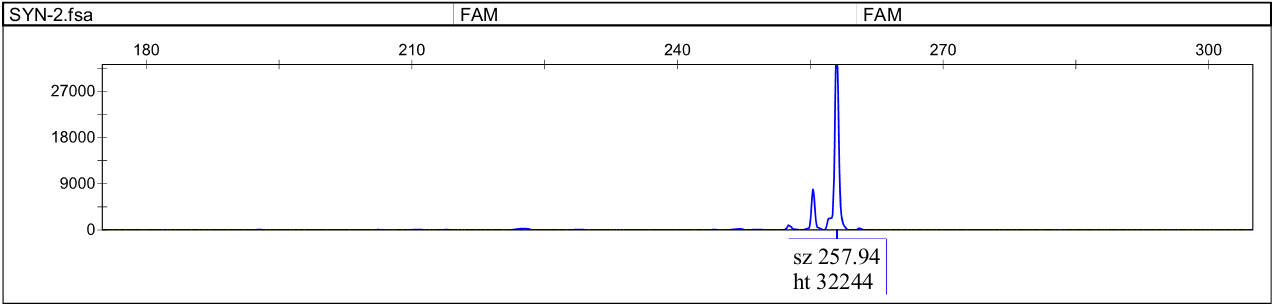


No.30


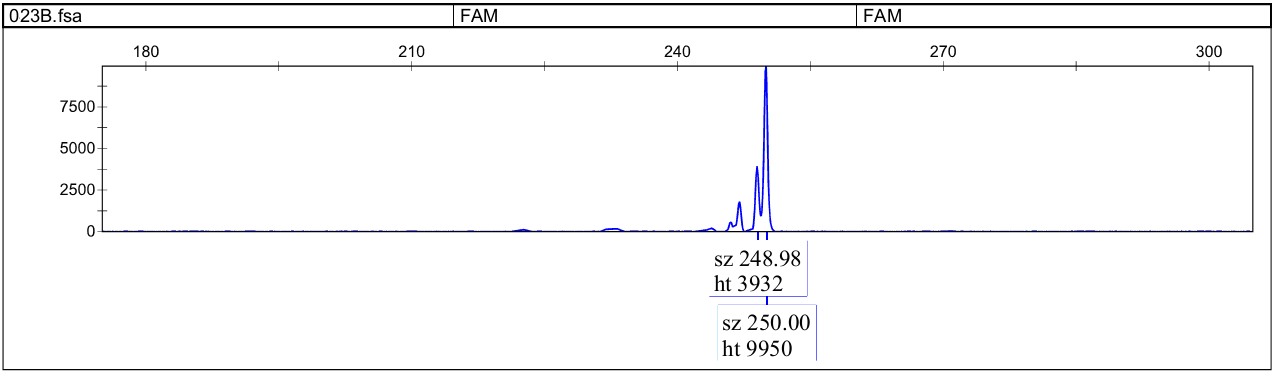


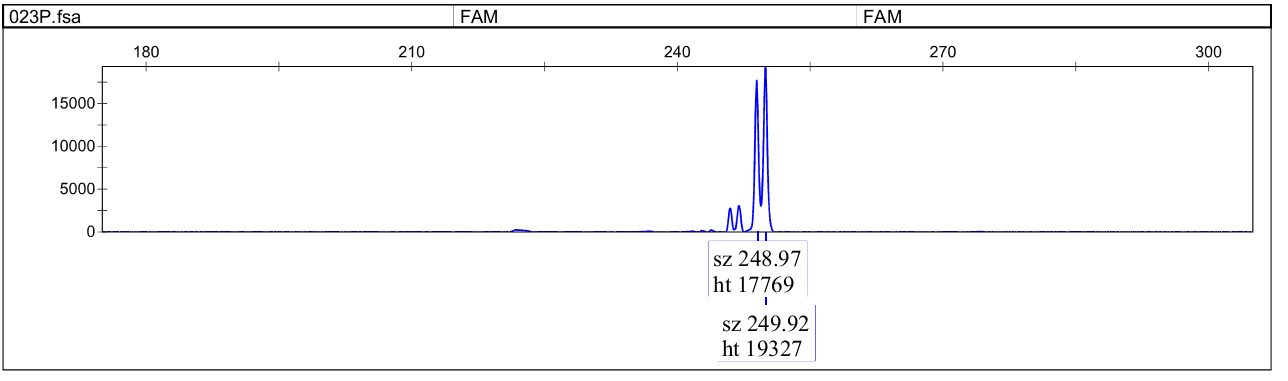


No.31


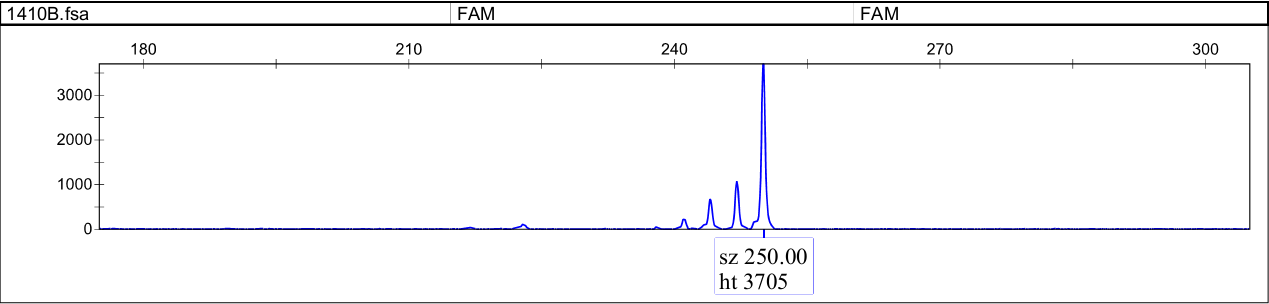


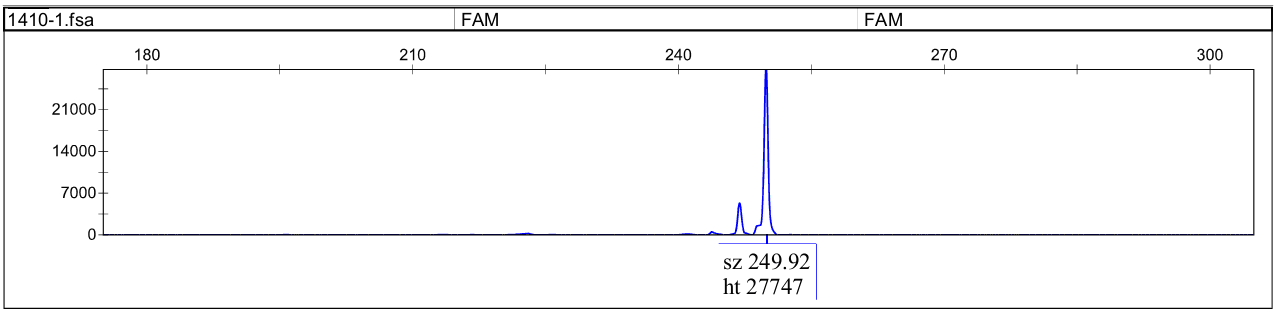

Supplement: Figure S1 — We detected CAG repeat length of bloods and tumor tissues for 31 MBC patients. The test results of two paired samples were the same in each patient. (DOC) [file pone.0052271.s001.doc]
